# PRAME and CTCFL-reactive TCRs for the treatment of ovarian cancer

**DOI:** 10.3389/fimmu.2023.1121973

**Published:** 2023-03-21

**Authors:** Rosa A. van Amerongen, Sander Tuit, Anne K. Wouters, Marian van de Meent, Sterre L. Siekman, Miranda H. Meeuwsen, Tassilo L. A. Wachsmann, Dennis F. G. Remst, Renate S. Hagedoorn, Dirk M. van der Steen, Arnoud H. de Ru, Els M. E. Verdegaal, Peter A. van Veelen, J. H. Frederik Falkenburg, Mirjam H. M. Heemskerk

**Affiliations:** ^1^ Department of Hematology, Leiden University Medical Center, Leiden, Netherlands; ^2^ Center for Proteomics and Metabolomics, Leiden University Medical Center, Leiden, Netherlands; ^3^ Department of Medical Oncology, Oncode Institute, Leiden University Medical Center, Leiden, Netherlands

**Keywords:** ovarian cancer, PRAME, CTCFL, CLDN6, TCR gene transfer, T-cell therapy, immunotherapy, allogeneic HLA

## Abstract

Recurrent disease emerges in the majority of patients with ovarian cancer (OVCA). Adoptive T-cell therapies with T-cell receptors (TCRs) targeting tumor-associated antigens (TAAs) are considered promising solutions for less-immunogenic ‘cold’ ovarian tumors. In order to treat a broader patient population, more TCRs targeting peptides derived from different TAAs binding in various HLA class I molecules are essential. By performing a differential gene expression analysis using mRNA-seq datasets, PRAME, CTCFL and CLDN6 were selected as strictly tumor-specific TAAs, with high expression in ovarian cancer and at least 20-fold lower expression in all healthy tissues of risk. In primary OVCA patient samples and cell lines we confirmed expression and identified naturally expressed TAA-derived peptides in the HLA class I ligandome. Subsequently, high-avidity T-cell clones recognizing these peptides were isolated from the allo-HLA T-cell repertoire of healthy individuals. Three PRAME TCRs and one CTCFL TCR of the most promising T-cell clones were sequenced, and transferred to CD8+ T cells. The PRAME TCR-T cells demonstrated potent and specific antitumor reactivity *in vitro* and *in vivo*. The CTCFL TCR-T cells efficiently recognized primary patient-derived OVCA cells, and OVCA cell lines treated with demethylating agent 5-aza-2′-deoxycytidine (DAC). The identified PRAME and CTCFL TCRs are promising candidates for the treatment of patients with ovarian cancer, and are an essential addition to the currently used HLA-A*02:01 restricted PRAME TCRs. Our selection of differentially expressed genes, naturally expressed TAA peptides and potent TCRs can improve and broaden the use of T-cell therapies for patients with ovarian cancer or other *PRAME* or *CTCFL* expressing cancers.

## Background

Ovarian cancer (OVCA) is the fifth most lethal cancer type among women (1). Due to lack of specific symptoms, 58% of the ovarian cancer patients are diagnosed at an advanced or metastatic stage. These advanced stages have 5-year survival rates of only 30%, compared to about 80% for earlier stages (2). Ovarian cancer is a heterogeneous malignancy, with five distinct histotypes of which high-grade serous ovarian cancer (HGSC) is the most frequent type covering 70% of all ovarian cancers (3). Although late-stage patients initially respond well to standard treatments like debulking surgery, platinum- and taxane-based chemotherapy, or more recently poly (ADP-ribose) polymerase inhibitors, recurrent disease emerges in the majority of patients (4–6). Also immunotherapies such as, infusion of tumor infiltrating lymphocytes (TILs), anti-cancer vaccination, treatment with immune checkpoint inhibitors, and adoptive T-cell therapies using chimeric antigen receptors (CARs) or T-cell receptors (TCRs) are being explored in ovarian cancer patients (7–9). CARs are restricted to target epitopes of proteins located at the cell membrane, with limited options for ovarian cancer. TCRs can target more antigens, since peptides derived from both intra- and extracellular proteins can be processed and presented in human leukocyte antigen (HLA) and thus recognized by TCRs.

Ovarian cancer is in general classified as an immunogenic tumor, with CD8+ T-cell rich tumors associating with prolonged survival (10–12). Furthermore, immune escape mechanisms correlate with poor survival, such as HLA downregulation and increased expression of immune inhibitory molecules (13). For T-cell infiltrated tumors (‘hot’ tumors), immune checkpoint inhibitors or infusion of TILs may be good strategies. However, in most ovarian tumors the tumor mutation burden (TMB) is low, resulting in limited T-cell infiltration, lack of antitumor-reactive T cells, and consequently ‘cold’ tumors (13, 14). For those ‘cold’ tumors, adoptive T-cell therapies with TCR-engineered T cells (TCR-T cells) targeting tumor-associated antigens (TAAs) are considered promising solutions (8). In clinical trials with ovarian cancer patients, TCRs targeting cancer-testis antigens (CTAs) NY-ESO-1, MAGE-A4 and more recently PRAME have been investigated (8). Preclinically, T cells targeting MSLN, CCNA1, CLDN6, and several MAGE-A family members have been investigated for ovarian cancer as well (15–18). Yet, targeting more TAAs is desired and target antigens restricted by more HLA alleles are essential, as most of the investigated TCRs are HLA-A*02:01 restricted. Ideal TAAs to target ovarian cancer would be those that are highly and homogenously expressed in tumors, without expression in healthy tissues. Co-expression in tissues from reproductive organs would be tolerable, as expression in the reproductive compartment does not form an unacceptable toxicity risk for ovarian cancer patients. In addition, protein expression or options to induce expression in case of variable expression are required. For example, DNA-demethylating agents have shown the potential to induce expression of some CTAs, thereby contributing to increased recognition by CTA-specific T cells (19–21). T cells targeting TAAs can be found in the T-cell repertoire of either healthy individuals or patients. If TAAs are also expressed in healthy tissues, self-tolerance is established during negative selection whereby high-avidity self-reactive T cells are centrally deleted from the autologous-HLA (auto-HLA) T-cell repertoire. Self-tolerance can be circumvented by searching for TAA-specific T cells in the allogeneic-HLA (allo-HLA) T-cell repertoire, as we previously demonstrated for several B-cell restricted antigens and WT1 (22–24). Since these T cells of the allo-HLA T-cell repertoire have not been subjected to negative selection, the safety should be carefully evaluated.

In order to treat a broader patient population, we searched for strictly tumor-specific TAAs in ovarian cancer and high-affinity TCRs targeting these TAAs. By combining mRNA-seq datasets of healthy and tumor tissues, we selected preferentially expressed antigen of melanoma (PRAME), CCCTC-binding factor (CTCFL), and Claudin-6 (CLDN6) as TAAs with high expression in ovarian cancer and at least 20-fold lower expression in all healthy tissues of risk. We identified peptides derived from the selected targets in the HLA class I ligandome of primary OVCA patient samples as well as cell lines. To target the identified peptides we isolated high-avidity T-cell clones from the allo-HLA T-cell repertoire of 25 healthy individuals. Using panels of primary patient-derived ovarian cancer cells, OVCA cell lines and healthy cell subsets, we ultimately selected three PRAME TCRs and one CTCFL TCR with potent and specific antitumor reactivity *in vitro* and *in vivo*. These TCRs are promising candidates for the treatment of patients with ovarian cancer.

## Materials and methods

### Differential gene expression analysis

Publicly available datasets [The Cancer Genome Atlas (TCGA) (https://www.cancer.gov/tcga); Genotype Tissue Expression (GTEx) (25); Human Protein Atlas (HPA) (26)] were accessed through the online resource Recount2 (https://jhubiostatistics.shinyapps.io/recount/) (27). Read alignment against the hg38 reference genome and mRNA quantification were part of the Recount2 pre-processing pipeline. Raw count tables were obtained and combined into one comprehensive dataset. For each distinct primary cancer tissue from the TCGA 30 samples were randomly chosen. Random sampling was also applied for the GTEx dataset, with maximum number of 20 samples, if available. Regarding the HPA dataset, all samples were included (3-5 samples per tissue). The compiled dataset consisted of a total of 2202 samples and was normalized utilizing the EdgeR package and its Relative Log Expression (RLE) method (28, 29) in R (v3.4.3). Finally, the dataset was filtered to retain only those genes showing evidence of expression in ovarian cancer, as defined by a minimum mean of 100 read counts (16855 genes in total). Differential gene expression analysis was performed using the EdgeR package after fitting a quasi-likelihood negative binomial generalized log-linear model to the count data. Genes were defined to be DE in ovarian cancer when they exhibited an absolute minimum fold change (FC) of ≥ 20 and FDR adjusted p-value of ≤ 0.05. Mean expression in ovarian cancer was compared against most of the healthy tissues present in the dataset, only tissues from reproductive organs and tumors were excluded.

### Sample collection for peptide elution

Seven solid primary OVCA patient samples derived from different patients (2 – 20 gram) were collected and dissociated using the gentleMACS (Miltenyi Biotec) procedure (**Supplemental Methods**). Also one ascites OVCA patient sample (6*10^9^ cells) and three primary acute myeloid leukemia (AML) samples (65 – 500*10^9^ cells) were collected. Furthermore, various cell lines were expanded up to at least 2*10^9^ cells (**Supplementary Table 3**). Cell lines transduced with HLA alleles, CLDN6 and/or CTCFL were first enriched for marker gene expression *via* magnetic-activated cell sorting (MACS) or fluorescence-activated cell sorting (FACS). HLA typing of all samples/cell lines was performed and gene expression was quantified by Quantitative Polymerase Chain Reaction (qPCR) (**Supplemental Methods**).

### HLA class I-peptide elution procedure, fractionation and mass spectrometry

Cell pellets were lysed and subjected to an immunoaffinity column to collect bound peptide-HLA complexes. Peptides were subsequently separated, fractionated and analyzed by data-dependent MS/MS (**Supplemental Methods**). Proteome Discoverer V.2.1 (Thermo Fisher Scientific) was used for peptide and protein identification, using the mascot search node for identification (mascot V.2.2.04) and the UniProt Homo Sapiens database (UP000005640; Jan 2015; 67,911 entries). Peptides were in-house synthesized using standard Fmoc chemistry and PE-conjugated pMHC-multimers were generated with minor modifications (**Supplemental Methods**).

### Cell culture

T cells were cultured in T-cell medium (TCM) and (re)stimulated every 10-14 days with PHA and irradiated autologous feeders (**Supplemental Methods**). OVCA cell lines COV-318/-362.4/-413b/-434/-504/-641 were established at the department of Medical Oncology (LUMC, NL) (30). OVCA cell lines OVCAR-3 and SK-OV-3 were obtained from the ATCC and A2780 from the ECACC. Primary patient-derived OVCA cells were either isolated from bulk tumor tissue using gentle MACS and immediately frozen (OVCA-L11) or isolated from the ascites fluid by centrifugation (>70% EpCAM positive cells and >95% CD45 negative cells) and immediately frozen (OVCA-L23). Both OVCA-L11 and OVCA-L23 were derived from an HLA-A*02:01 positive OVCA patient. The primary patient-derived OVCA cells (p0) were thawed three days before being used as target cells in screening experiments. Additionally, primary patient-derived OVCA-L23 cells expanded *in vitro* which allowed retroviral introduction of HLA-A*24:02 or B*07:01, followed by MACS-enrichment. OVCA-L23 cells transduced with HLA-A*24:02 or B*07:01 (passage 10) were included as target cells in screening experiments. Tumor cell lines and primary patient-derived OVCA cells were cultured in different media (**Supplemental Methods**). CD14-derived mature and immature dendritic cells (mDCs and imDCs), and activated CD19 cells were isolated from peripheral blood mononuclear cells (PBMCs) of different healthy donors and generated as previously described (24). Purity of the generated cells was assessed using flow cytometry (**Supplemental Methods**). Fibroblasts and keratinocytes, both cultured from skin biopsies, were cultured as previously described (24). PTECs derived from kidney tubules were isolated and cultured as previously described (31).

### Isolation of OVCA-specific T cells by pMHC-multimer enrichment

Buffy coats of healthy donors were collected after informed consent (Sanquin). PBMCs were isolated using Ficoll gradient separation and incubated with the selection of pMHC-multimers for 1 hour at 4°C or 15 minutes at 37°C. pMHC-multimers were only included if the healthy donor was negative for the restricted HLA allele. pMHC-multimer bound cells were MACS enriched using anti-PE MicroBeads (Miltenyi Biotec/130-048-801). The positive fraction was stained with CD8 (AF700) and CD4, CD14 and CD19 (FITC). pMHC-multimer and CD8 positive cells were single-cell sorted using an Aria III cell sorter (BD Biosciences) in a 96 well round bottom plate containing 5x10^4^ irradiated PBMCs (35Gy) and 5x10^3^ EBV-JY cells (55Gy) in 100 μL TCM with 0.8 µg/mL phytohemagglutinin (PHA). T-cell recognition was assessed 10 – 14 days after stimulation, followed by restimulation or storage of the selected T-cell clones.

### T-cell reactivity assays

T-cell recognition was measured by an IFN-γ ELISA (Sanquin or Diaclone). 5,000 T cells were cocultured overnight with target cells in various effector-to-target (E:T) ratios in 60 μL TCM in 384-well flat-bottom plates (Greiner Bio-One). To upregulate HLA expression, all adherent target cells were treated with 100 IU/mL IFN-γ (Boehringer Ingelheim) for 48 hours before coculture. All T cells and target cells were washed thoroughly before coculture to remove expansion-related cytokines. Supernatants were transferred during the ELISA procedure using the Hamilton Microlab STAR Liquid Handling System (Hamilton company) and diluted 1:5, 1:25 and/or 1:125 to quantify IFN-γ production levels within the linear range of the standard curve. T-cell mediated cytotoxicity was measured in a 6-hour ^51^chromium release assay (**Supplemental Methods**).

### TCR identification and TCR gene transfer to CD8+ T cells

TCR α and β chains of the selected T-cell clones were identified by sequencing with minor modifications (**Supplemental Methods**). The TCR α (VJ) and β (VDJ) regions were codon optimized, synthesized, and cloned in MP71-TCR-flex retroviral vectors by Baseclear. The MP71-TCR-flex vector already contains codon-optimized and cysteine-modified murine TCR α and β constant domains to optimize TCR expression and increase preferential pairing (32). Apart from the OVCA-specific TCRs, a murinized CMV-specific TCR (NLVPMVATV peptide presented in HLA-A*02:01) was included as a negative control. CD8+ T cells were isolated from PBMCs of different donors by MACS and TCRs were introduced *via* retroviral transduction two days after stimulation with PHA and irradiated autologous feeders. Seven days after stimulation, CD8+ T cells were MACS enriched for murine TCR. Ten days after stimulation, purity of TCR-T cells was checked by flow cytometry and used in functional assays (more details in **Supplemental Methods**).

### *In vivo* model

NOD-scid-IL2Rgamma^null^ (NSG) mice (The Jackson Laboratory) were intravenously (i.v.) injected with 2*10^6^ U266 multiple myeloma (MM)_cells. U266 cells were transduced with and enriched for Luciferase-tdTomato Red and HLA-A24 (NGFR) when indicated. On day 14, mice were treated i.v. with 5*10^6^ purified PRAME TCR-T cells (n = 6) or CMV TCR-T cells (n = 4). TCR-T cells were used seven days after second stimulation with PHA and irradiated autologous feeder cells. Tumor outgrowth (average radiance) was measured at regular intervals after intraperitoneal injection of 150 mL 7.5 mM D-luciferine (Cayman Chemical) using a CCD camera (IVIS Spectrum, PerkinElmer). All mice were sacrificed when control mice reached an average luminescence of 1*10^7^ p/s/cm^2^/sr. This study was approved by the national Ethical Committee for Animal Research (AVD116002017891) and performed in accordance with Dutch laws for animal experiments.

### DAC treatment

DAC (5-aza-2′-deoxycytidine) (A3656, Sigma-Aldrich) was solved in dimethyl sulfoxide (DMSO). Target cells were at 50% confluency at start of treatment and were treated with 1 µM DAC on day 1 and 4. DMSO treated cells served as negative control. On day 7, cells were harvested for T-cell reactivity assays and RNA isolation to determine gene expression by qPCR.

### Statistical analysis

Statistical analysis was performed using GraphPad Prism software (Version 9.0.1.). Statistical tests used are indicated in the figure legends, P < 0.05 was considered significant. Significance levels are indicated as p <.05 *, p <.01 **, p <.001 ***, and p <.0001 ****.

### Study approval

Samples of healthy donors and AML patients were used from the LUMC Biobank for Hematological Diseases, after approval by the Institutional Review Board of the LUMC (approval number 3.4205/010/FB/jr) and the METC-LDD (approval number HEM 008/SH/sh). The OVCA patient samples were obtained according to the Code of Conduct for Responsible Use of human tissues or in the context of study L18.012 that was approved by the Institutional Review Board of the LUMC (approval number L18.012) and Central Committee on Research Involving Human Subjects (approval number NL63434.000.17). Studies were conducted in accordance with the Declaration of Helsinki and after obtaining informed consent.

## Results

### Interrogation of mRNA-seq data reveals differentially expressed genes in ovarian cancer

**Figure 1:**
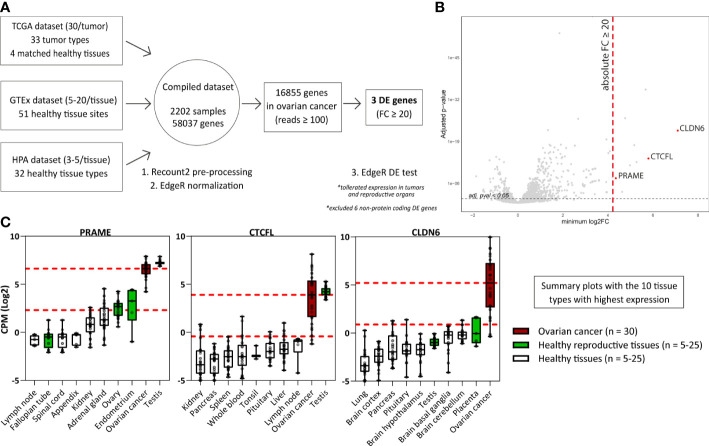
Differential gene expression analysis reveals genes associated with High-Grade Serous Ovarian Carcinoma. **(A)** Scheme depicting the differential gene expression analysis strategy. **(B)** Plot displaying for all genes the minimum FC against the adj. p-val. Indicated in red are the three identified DE genes (FC ≥ 20; adj. p-val ≤ 0.05). Indicated in grey are non-DE genes and non-protein coding genes. **(C)** Boxplots depicting *PRAME*, *CTCFL* and *CLDN6* expression in ovarian cancer (TCGA data, n = 30) and the 9 healthy tissue types with highest gene expression (HPA and/or GTEx data, n = 5-25). Overlapping healthy tissue types within the HPA and GTEx were combined when possible. Boxplots extend from first to third quartile, the horizontal line represent the median expression value. The whiskers represent minimum and maximum expression. The upper and lower red dashed lines represent the median expression value and the 20 times lower expression value, respectively. (Adj. p-val: false discovery rate adjusted p-value, DE, differentially expressed; FC, fold change; GTEx, genotype-tissue expression; HPA, human protein atlas; CPM Log2, log2-transformed counts per million; minimum log2FC, log2 fold change; TCGA, The cancer genome atlas).

To identify genes with immuno-therapeutic potential in ovarian cancer, we obtained mRNA-seq data of 2202 samples from three independent sources (TCGA, GTEx, and HPA) representing 120 different healthy or tumor tissues. We combined these tissues into one comprehensive dataset to perform an elaborate differential gene expression analysis (**Figure 1A** and **Supplementary Table 1**). Genes were defined to be differentially expressed (DE) in ovarian cancer when they exhibited an absolute FC of ≥ 20 compared to the different healthy tissues present in the dataset, using the mean expression values. Tissues from reproductive organs were excluded from this comparison, as expression in the reproductive compartment does not form an unacceptable toxicity risk for ovarian cancer patients. The FC values for all 16,855 genes with ≥ 100 read counts in ovarian cancer are listed in **Supplementary Table 2**, of which 9 genes were DE with a FC ≥ 20 in ovarian cancer. We plotted for all genes the minimum FC against the adjusted p-value to visualize the minimal extent of differential expression in ovarian cancer (**Figure 1B**).

Six of the nine DE genes are not expressed on protein level and were therefore not considered target candidates for T-cell therapy. SLC25A3P1, small nuclear RNU1-27P and small nuclear RNU1-28P are pseudogenes which are assumed not to be translated (33). Furthermore, microRNA MIR3687-1, antisense RNA ELFN1-AS1 and an uncharacterized long non-coding RNA gene are classified as non-protein coding RNAs, although they do exhibit several gene regulating functions of other genes (34). The final three genes, PRAME, CTCFL and CLDN6, were considered interesting target candidates. These genes were at least 20 times higher expressed in ovarian cancer compared with healthy tissues, except for some reproductive organs (**Supplementary Figure 1**, summarized in **Figure 1C**). In line with their classification as CTA, *PRAME* and *CTCFL* were highly expressed in testis (35). *PRAME* was also found to be expressed in healthy endometrium and ovary, and *CLDN6* in placenta. According to the TCGA data, in particular *PRAME* is expressed in various other tumor types as well (**Supplementary Figure 2**).

**Figure 2:**
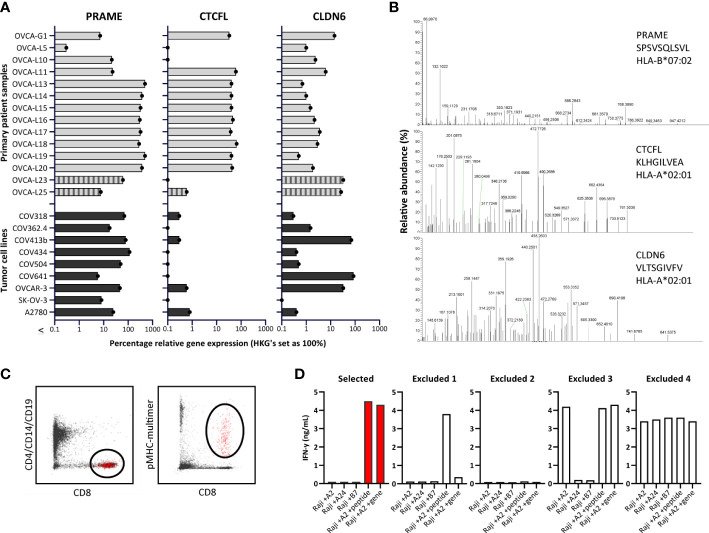
Identification of PRAME, CTCFL and CLDN6 peptides and T-cell clones. **(A)** PRAME, CTCFL (TvX) and CLDN6 mRNA gene expression in 14 OVCA patient samples (12 solid tumor tissues and 2 malignant ascites samples (OVCA-L23 and OVCA-L25)), and 9 OVCA cell lines. Expression was measured by qPCR and is shown as percentage relative to the three HKGs *GUSB*, *VPS29* and *PSMB4*, which was set at 100%. **(B)** Example of three OVCA-derived peptides identified in our HLA ligandome analyses. Shown are the mass spectra of the eluted peptides, including the gene, peptide sequence and HLA restriction. All eluted peptides were validated by comparing tandem mass spectra of eluted peptides and synthetic peptides, as shown in **Supplementary Figure 3**. **(C)** Representative flow cytometry plots of the pMHC-multimer enriched cell population in 1 of the 25 healthy donors. Shown is the gating strategy of the single-cell sorted population (depicted in red), gated on CD8 (Alx700) +, pMHC-multimer (PE) + and CD4/CD14/CD19 (FITC) -. **(D)** Examples of recognition patterns based on IFN-γ production (ng/mL) of selected and excluded T-cell clones during the first T-cell screenings. T-cell clones were cocultured with Raji cells transduced with various HLA alleles, combined with loading of OVCA peptides (1 μM) or transduction of OVCA genes (E:T=1:6). Excluded 1 – 4 represent T-cell clones lacking potency and/or specificity. (HKGs, housekeeping genes; OVCA, primary ovarian cancer sample).

To confirm expression of the three selected genes in ovarian cancer, we quantified gene expression by qPCR in primary solid tumor patient samples and malignant ascites patient samples, and in OVCA cell lines (**Figure 2A**). We quantified relative gene expression compared with three housekeeping genes. *PRAME* and *CLDN6* expression was demonstrated in most primary patient samples and OVCA cell lines. Expression of *CTCFL* was high (>30% relative expression) in 10/12 solid tumor patient samples, but limited expression was observed in ascites patient samples and cell lines.

### PRAME, CTCFL and CLDN6-derived peptides identified in the HLA class I ligandome

The number of previously identified peptides derived from PRAME, CTCFL and CLDN6 binding in different common HLA class I molecules is limited, as well as solid evidence of processing and presentation in the context of HLA class I on ovarian tumors. The PRAME TCRs currently investigated in clinical trials all target the SLLQHLIGL or VLDGLDVLL peptide presented in HLA-A*02:01. To establish a dataset of peptides that can be targeted by TCRs, we determined the HLA class I ligandome of eight primary OVCA patient samples and two OVCA cell lines (**Supplementary Tables 3A, B**). In order to enlarge the dataset, various tumor cell lines and primary AML patient samples expressing the selected genes were additionally included (**Supplementary Table 3C**), some of these cell lines were transduced with CTCFL, CLDN6 and/or HLA class I molecules (**Supplementary Table 3D**). All best scoring peptides for each gene with preferably a minimal Best Mascot Ion score of 20 and a mass accuracy of 10 ppm were considered in the first round of selection. As CLDN6 and CTCFL share homology with ubiquitously expressed family members, only those peptides that were unique for the target genes and did not demonstrate major sequence overlap with Claudin-family members (n=47) or paralog CTCF (n=6) were selected. In addition, we only continued with peptides binding to common HLA molecules according to netMHC peptide binding algorithm that matched with the HLA typing of the material from which the peptides originated (**Supplementary Tables 3A–D**) (36). Identified peptides were validated by comparing mass spectra of eluted peptides and synthetic peptides (**Figure 2B** and **Supplementary Figure 3**). HLA binding was confirmed by stable pMHC-monomer refolding. In total 23 PRAME peptides, 8 CTCFL peptides and 3 CLDN6 peptides were validated (**Supplementary Table 4**). As a result of alternative splicing, at least 15 protein variants derived from CTCFL isoforms are known (37). 7/8 CTCFL peptides are present in all 15 CTCFL variants, 1/8 CTCFL peptides, KLHGILVEA in HLA-A*02:01, is only located in the unique region of CTCFL variant 13 (**Supplementary Figures 4A, B**) (37). Since no substantial differences in gene expression were observed between variant 13 and the other CTCFL variants we also continued with this peptide (**Supplementary Figure 4C**).

### OVCA-reactive T-cell clones isolated from the allo-HLA T-cell repertoire of 25 healthy donors

To isolate high-avidity T cells reactive against PRAME-, CTCFL- and CLDN6-derived peptides, peptide MHC-multimers (pMHC-multimers) were generated for a selection of 17 peptides binding in different common HLA class I alleles (**Table 1**). Of these peptides 16 were identified in our mass spectrometry analysis and 1 peptide was previously identified (38). These pMHC-multimers were incubated with PBMCs of 25 healthy HLA typed donors, pMHC-multimer+ cells were enriched by MACS, and pMHC-multimer+ CD8+ cells were subsequently single-cell sorted (**Figure 2C**). pMHC-multimers were only included if the donor was negative for the HLA allele, to ensure identification of T cells from the allogeneic T-cell repertoire, and thereby circumventing self-tolerance. On average 618*10^6^ PBMCs were used per donor and between 21 and 368 pMHC-multimer+ CD8+ T-cell clones could be expanded after single-cell sorting. To test for functional peptide-specificity, T-cell clones were cocultured with Raji cells loaded with a pool of all target peptides. T-cell clones specifically recognizing the peptide pool were subsequently tested for recognition of target cells transduced with OVCA genes, to select T-cell clones potent enough to recognize endogenously processed and presented peptide. T-cell clones that were only reactive against peptide-loaded cells, nonreactive, reactive against one specific HLA allele independent of added peptides, or reactive against all target cells were excluded (**Figure 2D**). In addition to our search in healthy donors, we searched within the allogeneic T-cell repertoire of an AML patient after HLA-mismatched stem cell transplantation that was published previously (39).

**Table 1:** Included PRAME, CTCFL and CLDN6 HLA class I peptides.

Gene	Peptide	HLA	Sample/cell line source	BMI
PRAME	QLLALLPSL	A*02:01	TMD8 +A2, EBV-5098	37
PRAME	LYVDSLFFL	A*24:02	x	x
PRAME	SPRRLVELAGQSL	B*07:02	COV413b, AML-6711, TMD8 +B7, EBV-5098	30
PRAME	MPMQDIKMIL	B*07:02	TMD8 +B7, AML-6498	25
PRAME	SPSVSQLSVL	B*07:02	COV413b, EBV-5098, TMD8 +B7, AML-3374, U266	65
PRAME	LPRELFPPL	B*07:02	EBV-5098, K562+B7	26
PRAME	MPMQDIKMIL	B*35:01	TMD8 +B7, AML-6498	25
PRAME	LPRELFPPL	B*35:01	EBV-5098, K562+B7	26
PRAME	YEDIHGTLHL	B*40:01	COV362.4, U266	42
CTCFL	CSAVFHERY	A*01:01	K562+A1	43
CTCFL	RSDEIVLTV	A*01:01	K562+A1	37
CTCFL	KLHGILVEA	A*02:01	K562+A2	12
CTCFL	DSKLAVSL	B*08:01	K562+B8	35
CTCFL	AETTGLIKL	B*40:01	COV362.4	51
CLDN6	GPSEYPTKNYV	A*01:01	EBV-9603 +CLDN6	25
CLDN6	VLTSGIVFV	A*02:01	EBV-6519 +CLDN6	23
CLDN6	DSKARLVL	B*08:01	EBV-9603 +CLDN6	37

Overview of the 17 OVCA gene-derived peptides included in our T-cell search. For each peptide identified in our HLA ligandome analyses, the gene, HLA binding restriction, sample/cell line source, and BMI are listed. Details of the samples and cell lines are listed in **Supplementary Table 1**. The LYVDSLFFL peptide binding in A*24:02 was included based on literature (38). BMI, best Mascot ion score.

In total, 56 T-cell clones specific for 6/9 PRAME and 3/5 CTCFL peptides that recognized cells transduced with the respective OVCA gene were selected of which 28 clones are shown in **Supplementary figure 5A, B**. For CLDN6, T-cell clones were isolated that recognized peptide-loaded target cells (**Supplementary figure 5C**), however, CLDN6 transduced cells were not recognized and therefore these CLDN6-specific T-cell clones were not of sufficient avidity and excluded from further screenings.

### T-cell clones selected as clinical TCR candidates for the treatment of ovarian cancer patients

**Figure 3:**
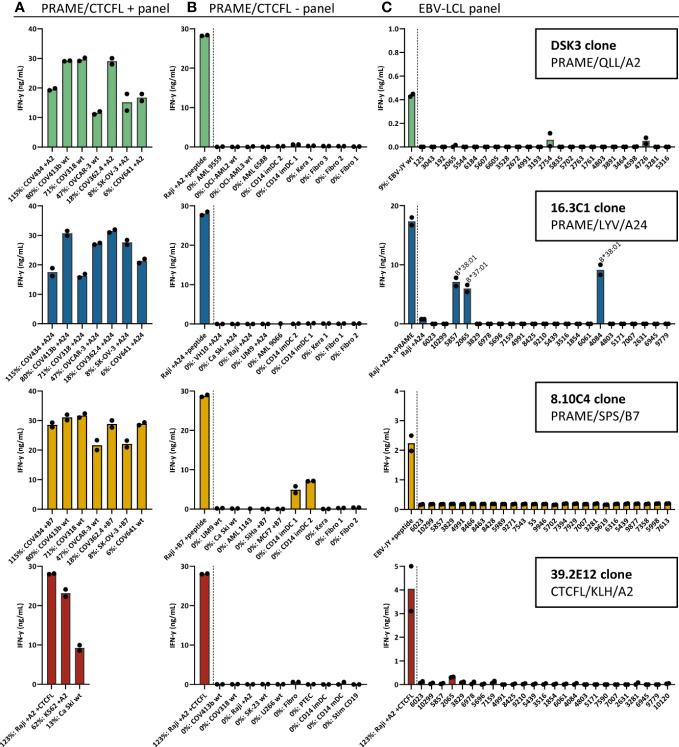
Recognition patterns of the selected T-cell clones recognizing *PRAME* or *CTCFL* positive tumor cells, without substantial peptide or HLA cross-reactivity. Recognition patterns based on IFN-γ production (ng/mL) after overnight coculture assays with **(A)** *PRAME* or *CTCFL* positive tumor cell lines, **(B)** PRAME or CTCFL-negative tumor cell lines and healthy cell subsets, and **(C)** 25 EBV-LCLs, expressing all HLA alleles with an allele frequency ≥ 1% present in the Caucasian population. The HLA allele in **(C)** is depicted if an HLA allele is recognized by the T-cell clone, meeting the requirement that all EBV-LCLs with this HLA allele are recognized. All cell lines in **(A, B)** express the HLA allele that presents the targeted peptide, either wildtype or the HLA allele was introduced by transduction (+A2, +A24 or +B7). Percentage relative *PRAME* or *CTCFL* expression is depicted, as determined by qPCR. Bars represent mean and symbols depict technical duplicates. (EBV-LCL: Epstein-Barr virus transformed lymphoblastoid cell lines).

To select TCR candidates for clinical development, 3 additional screenings were performed. First, tumor recognition was assessed using a panel of naturally expressing *PRAME* or *CTCFL* positive tumor cell lines, all expressing the target HLA allele. OVCA cell lines were included to screen the PRAME T-cell clones and for the CTCFL T-cell clones K562 and Ca Ski cell lines were included since OVCA cell lines did not express *CTCFL* (**Figure 2A**). Second, cross-reactivity with other peptides presented in the target HLA allele was assessed using a panel of *PRAME* or *CTCFL* negative tumor cell lines and healthy cell subsets. Third, HLA cross-reactivity was assessed using a panel of Epstein-Barr virus transformed lymphoblastoid cell lines (EBV-LCL) expressing all HLA alleles with an allele frequency ≥ 1% present in the Caucasian population. In total, four T-cell clones were selected as TCR candidates for clinical development. Three T-cell clones target a PRAME-derived peptide: clone DSK3 specific for QLLALLPSL in HLA-A*02:01 (PRAME/QLL/A2), clone 16.3C1 specific for LYVDSLFFL in HLA-A*24:02 (PRAME/LYV/A24) and clone 8.10C4 specific for SPSVSQLSVL in B*07:02 (PRAME/SPS/B7). One T-cell clone targets a CTCFL-derived peptide: clone 39.2E12 specific for KLHGILVEA in HLA-A*02:01 (CTCFL/KLH/A2). These T-cell clones effectively recognized all *PRAME* or *CTCFL* positive OVCA/tumor cell lines (**Figure 3A**). Of the *PRAME* and *CTCFL* negative cells, only clone 8.10C4^PRAME/SPS/B7^ showed low recognition of *PRAME* negative healthy imDCs (**Figure 3B**). To prevent unwanted toxicity, this recognition should be investigated further using TCR-T cells. Furthermore, clone 16.3C1^PRAME/LYV/A24^ showed cross-reactivity against HLA-B*37:01 and HLA-B*38:01 positive EBV-LCLs (**Figure 3C**). The global frequencies of these HLA alleles are low (HLA-B*37:01: 3.23% and HLA-B*38:01: 1.72%) (40). The excluded T-cell clones exhibited either limited recognition of *PRAME* or *CTCFL* positive OVCA/tumor cell lines (25/56), or were cross-reactive against peptides in commonly expressed HLA alleles (27/56).

### High-affinity PRAME TCRs reactive against OVCA cells

**Figure 4:**
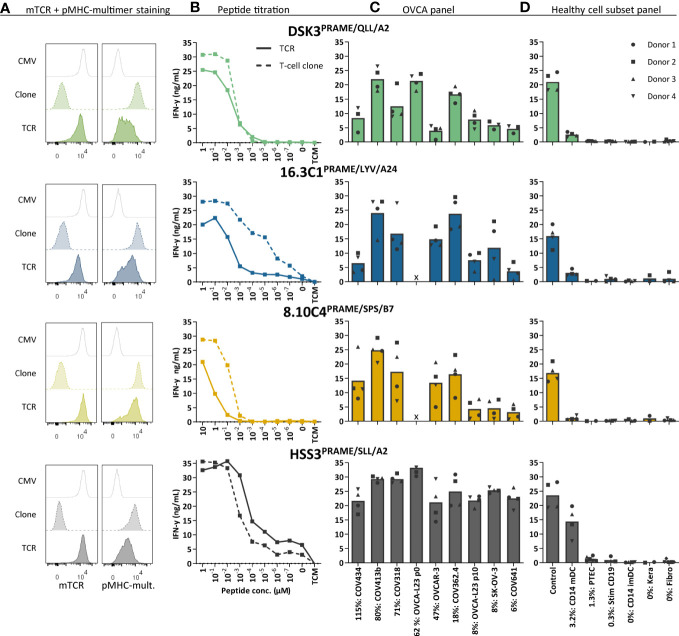
Three new PRAME TCR-T cells recognize *PRAME* positive OVCA cells and mature DCs. The three new PRAME TCRs and clinically tested HSS3 TCR were introduced *via* retroviral transduction in CD8+ cells of four different donors. **(A)** Representative flow cytometry plots of purified CMV and PRAME TCR-T cells, and their parental PRAME T-cell clones stained with murine TCR (mTCR) and the PRAME-specific pMHC-mult. **(B)** IFN-γ production (ng/mL) of TCR-T cells and their parental T-cell clones cocultured overnight with Raji cells (transduced with HLA-A2, A24 or B7) loaded with titrated peptide concentrations (E:T = 1:6). **(C)** IFN-γ production of TCR-T cells cocultured with OVCA cells (E:T = 1:6). All OVCA cells express the HLA allele that presents the targeted peptide, either wildtype or the HLA allele was introduced by transduction. Primary malignant ascites patient sample OVCA-L23 (wildtype HLA-A2) was either passage 0 (included for TCR DSK3 and HSS3) or passage 10 transduced with HLA-A24 or B7 (included for all TCRs). **(D)** IFN-γ production of TCR-T cells cocultured with several healthy cell subsets (E:T = 1:4 for keratinocytes, fibroblasts, PTECs and CD14+, 1:6 for CD19+). Cell subsets were isolated from multiple HLA-A2+, A24+ and/or B7+ donors. **(C-D)** Percentage relative *PRAME* expression is depicted, as determined by qPCR. Bars represent mean and symbols depict averaged duplicate values from four different donors tested in two independent experiments. (E:T, effector:target ratio; imDCs and mDCs, immature and mature dendritic cells; pMHC-mult, peptide MHC-multimers; PTECs, proximal tubular epithelial cells; OVCA, primary ovarian carcinoma sample).

To investigate the clinical potential of the selected PRAME T-cell clones for TCR gene therapeutic strategies, the TCR α and β chains were sequenced and transferred using retroviral vectors into CD8+ T cells of at least four different donors. TCR-T cells were enriched based on murine TCR (mTCR) expression and functionally tested. In **Figure 4A** we demonstrated, by pMHC-multimer staining, that PRAME TCR-T cells efficiently expressed the three newly identified TCRs at the cell surface. As a reference, the previously identified HSS3 TCR^PRAME/SLL/A2^ (patent: WO2016142783A2) that will be clinically tested in the near future was included (39). Most TCR-T cells exhibited high peptide sensitivity in peptide titration experiments, only TCR 8.10C4^PRAME/SPS/B7^ demonstrated limited peptide sensitivity (**Figure 4B**). Additionally, ovarian cancer reactivity of the different PRAME TCR-T cells was studied against various OVCA tumor cell lines and primary patient-derived ovarian cancer cells (OVCA-L23) (**Figure 4C**). The OVCA-L23 cells positive for HLA-A*02:01 expanded *in vitro* which allowed additional retroviral introduction of HLA-A*24:02 or B*07:01. Uncultured OVCA-L23 (p0) cells were therefore included as target for TCR DSK3^PRAME/QLL/A2^ and HLA-A*24:02 or B*07:01 transduced cells (p10) were included as targets for all the PRAME TCR-T cells. All PRAME TCR-T cells recognized the primary patient-derived OVCA-L23 cells as well as all seven PRAME positive OVCA tumor cell lines. In addition, the specificity of the PRAME TCR-T cells was tested against various healthy cell subsets. By qPCR relative *PRAME* expression was observed in mDCs (3.2%), PTECs (1.3%) and stimulated CD19 cells (0.3%) (**Supplementary Figure 6**). mDCs were slightly recognized by the PRAME TCRs, as was previously observed for the HSS3 TCR^PRAME/SLL/A2^ (39), but no other reactivity was observed (**Figure 4D**). Although clone 8.10C4^PRAME/SPS/B7^ had exhibited some reactivity against imDCs (**Figure 3B**), the TCR-T cells did not show any signs of recognition in repeated experiments (**Figure 4D**).

**Figure 5:**
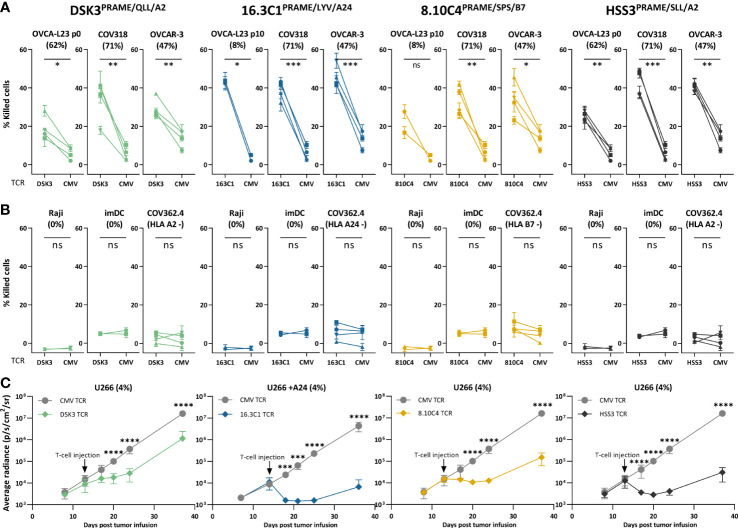
PRAME TCR-T cells kill OVCA cells *in vitro* and demonstrate *in vivo* killing potential in an established MM model. **(A, B)** Purified PRAME TCR-T cells were tested for cytotoxic capacity in a 6-hour ^51^Cr-release assay at E:T ratio 10:1 against **(A)** primary OVCA patient samples and OVCA cell lines, and **(B)** *PRAME* negative cells (Raji and imDCs), or target HLA negative cells (COV362.4). Except for COV362.4, all target cells expressed the target HLA alleles, either wildtype or Td. COV318 and OVCAR-3 were Td with A24, Raji cells were Td with A2, A24 or B7. Primary malignant ascites patient sample OVCA-L23 (wildtype HLA-A2) was either passage 0 (included for TCR DSK3 and HSS3) or passage 10 Td with A24 or B7 (included for TCR 16.3C1 and 8.10C4). imDCs were isolated from PBMCs of a A2+, A24+ and B7+ donor. Percentage relative *PRAME* expression is depicted, as determined by qPCR. Cytotoxic capacity of PRAME TCR- and CMV TCR-T cells were compared using a paired t-test (two-sided). Mean and SD of technical triplicates are depicted for four donors tested in two independent experiments. **(C)** NSG mice engrafted with 2*10^6^ U266 MM cells Td with *Luc2* luciferase. Mice were i.v. treated with 5*10^6^ PRAME or CMV TCR-T cells 14 days after tumor infusion. Mean and SD of tumor outgrowth (average radiance measured by bioluminescence imaging) over time on the ventral side are depicted. N=6 for PRAME TCR-T cells and n=4 for CMV TCR-T cells. Tumor outgrowth in mice treated with PRAME or CMV-TCR T cells was compared for each time point using two-way ANOVA on log-transformed data, followed by Bonferroni *post-hoc* analysis. Only significant results are depicted. (ANOVA, analysis of variance, E:T, effector:target ratio; ns, not significant; imDCs, immature dendritic cells; MM, multiple myeloma; OVCA, primary ovarian carcinoma sample; Td, transduced). Meaning of the * are listed in the M&M. Significance levels are indicated as p <.05 *, p <.01 **, p <.001 ***, and p <.0001 ****. ns, not significant.

Anti-OVCA cytotoxic reactivity was further investigated in a six-hour ^51^chromium release assay. Transfer of the different PRAME TCRs to CD8+ T cells of four different donors resulted in efficient killing of OVCA tumor cell lines and the primary patient-derived OVCA cells (OVCA-L23 p0 or p10) (**Figure 5A**). Comparable killing percentages were observed by positive control TCR HSS3^PRAME/SLL/A2^ (**Figure 5A**), and peptide-loaded targets were similarly lysed (**Supplementary Figure 7**). No off-target killing of Raji cells (0% *PRAME*), imDCs (0% *PRAME*), and target HLA negative COV362.4 cells was observed (**Figure 5B**). *In vivo* killing potential of the PRAME TCRs was tested in an established model for multiple myeloma (MM) (23), since *PRAME* is also expressed in MM. Despite low *PRAME* expression (4%), all three newly identified PRAME TCR-T cells and positive control TCR HSS3^PRAME/SLL/A2^ reduced tumor burden for at least 6 days after infusion (**Figure 5C**). TCR 16.3C1^PRAME/LYV/A24^ and the positive control demonstrated the strongest effect. In conclusion, the three PRAME TCRs (DSK3^PRAME/QLL/A2^, 16.3C1^PRAME/LYV/A24^ and 8.10C4^PRAME/SPS/B7^) demonstrated potent antitumor reactivity *in vitro* and *in vivo* without harming healthy cell subsets *in vitro* and are considered promising TCRs for TCR gene therapy.

### High-affinity CTCFL TCR reactive against DAC treated OVCA cells

**Figure 6:**
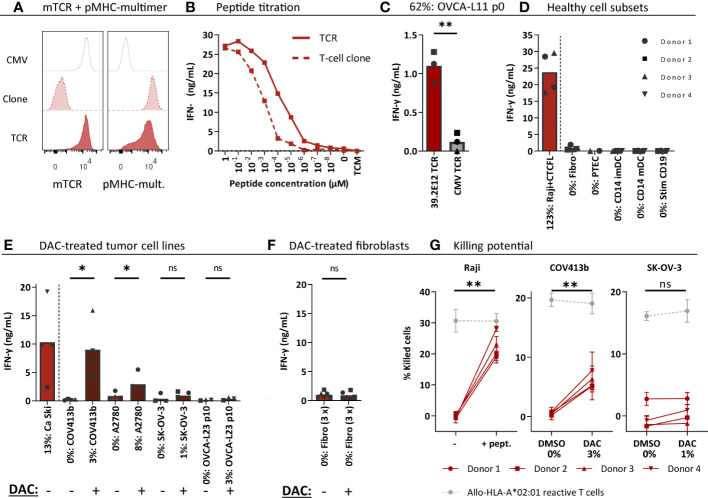
CTCFL TCR-T cells recognize and kill (DAC-treated) *CTCFL* positive OVCA cells. CD8+ cells of four different donors were retrovirally transduced to express the 39.2E12^CTCFL/KLH/A2^ TCR and purified. **(A)** Representative flow cytometry plots of purified CMV and CTCFL TCR-T cells, and the parental CTCFL T-cell clone stained with murine TCR (mTCR) and the CTCFL-specific pMHC-mult. **(B)** IFN-γ production (ng/mL) of the TCR-T cells and parental T-cell clone cocultured overnight with Raji cells transduced with HLA-A*02:01 and loaded with titrated peptide concentrations (E:T = 1:6). **(C–F)** IFN-γ production of TCR-T cells cocultured with **(C)** single viable cells of primary patient-derived sample OVCA-L11 passage 0 (E:T 1:6), **(D)** healthy cell subsets of multiple donors (E:T = 1:4 for fibroblasts, PTECs and CD14+, and 1:6 for CD19+), **(E)** 7 days 1 µM DAC or DMSO treated tumor cells (E:T = 1:6), and **(F)** 7 days 1 µM DAC or DMSO treated fibroblasts. Bars represent mean and symbols depict averaged duplicate values from three or four different donors tested in two independent experiments. **(G)** Cytotoxic capacity of CTCFL TCR-T cells in a 6-hour ^51^Cr-release assay against Raji cells loaded with the KLH peptide, and COV413b and SK-OV-3 treated with 7 days 1 µM DAC or DMSO. Mean and SD depict technical triplicates from four different donors tested in two independent experiments, at E:T ratio 10:1. Cytotoxic capacity of an allo-HLA-A*02:01 reactive T-cell clone recognizing HKG USP11 is shown for the different conditions (41). **(B–G)** All target cells express HLA-A*02:01, either wildtype or the HLA allele was introduced by transduction (Raji, SK-OV-3, A2780). Percentage relative *CTCFL* (TvX) expression is depicted, as determined by qPCR. **(D)** IFN-γ production of CTCFL TCR- and CMV TCR-T cells compared using a paired t-test (two-sided). **(E–G)** IFN-γ production and cytotoxicity of CTCFL TCR-T cells cocultured with DMSO and DAC-treated cells, or Raji cells loaded with and without peptide, compared using a paired t-test (two-sided). (ns, not significant; DAC, 5-aza-2′-deoxycytidine; imDCs and mDCs, immature and mature dendritic cells; pMHC-mult, peptide MHC-multimers; PTECs, proximal tubular epithelial cells; OVCA, primary ovarian carcinoma sample). Meaning of the * are listed in the M&M. Significance levels are indicated as p <.05 *, p <.01 **. ns, not significant.

Next, the CTCFL-specific TCR 39.2E12^CTCFL/KLH/A2^ was tested for anti-ovarian cancer reactivity and specificity. Generated CTCFL TCR-T cells efficiently expressed the TCR at the cell surface (**Figure 6A**) and demonstrated high peptide sensitivity in a peptide titration (**Figure 6B**). CTCFL TCR-T cells generated from three different donors recognized primary patient-derived OVCA-L11 cells harvested from an HLA-A*02:01 positive patient (**Figure 6C**). Furthermore, in line with the lack of *CTCFL* expression in any of the included healthy cell subsets (**Supplementary Figure 6**), healthy cell subsets were not recognized by CTCFL TCR-T cells (**Figure 6D**).

Despite high *CTCFL* expression in primary OVCA patient samples, OVCA tumor cell lines did not express *CTCFL* (**Figure 2A**). In contrast, cervical cancer cell line Ca Ski is positive for CTCFL and this correlates with expression in part of primary cervical carcinoma samples (**Supplementary Figure 2B**). As demonstrated in **Figure 6E**, the Ca Ski cells were efficiently recognized by the CTCFL TCR-T cells. Since *CTCFL* expression is epigenetically regulated and treatment with demethylating agent DAC has previously been shown to upregulate expression of *CTCFL* in OVCA tumor cell lines (42), we investigated whether DAC can make tumor cell-lines more susceptible to CTCFL-mediated killing. Seven days of DAC treatment clearly resulted in *CTCFL* upregulation in OVCA cell lines, compared to not treated cells (**Figure 6E**). In line with the upregulation, recognition of DAC-treated OVCA tumor cell lines COV413b and A2780 was significantly increased for CTCFL TCR-T cells (**Figure 6E**). *CTCFL* upregulation by DAC was restricted to tumor cells, as DAC treatment of healthy fibroblasts did not upregulate *CTCFL* expression and did not induce recognition by CTCFL TCR-T cells (**Figure 6F**). In line with increased cytokine production, cytotoxic capacity of CTCFL TCR-T cells towards DAC-treated COV413b was significantly increased (**Figure 6G**). DAC treatment did not increase killing by allo-HLA-A*02:01 T cells (**Figure 6G**) neither did it influence killing of peptide-loaded target cells (**Supplementary Figure 8**), suggesting DAC treatment does not generally increase susceptibility of these target cells to T-cell mediated killing. In OVCA tumor cell lines we also observed increased *PRAME* expression after DAC treatment, which slightly increased recognition and killing potential by HLA-A*02:01-restricted PRAME TCR-T cells (**Supplementary Figure 9**). In conclusion, CTCFL-specific TCR 39.2E12^CTCFL/KLH/A2^ demonstrate anti-OVCA reactivity against (DAC-treated) *CTCFL* positive tumor cells without harming healthy cell subsets and is considered a promising TCR for TCR gene therapy of ovarian cancer.

## Discussion

In this study, we describe the selection of PRAME, CTCFL and CLDN6 as strictly tumor-specific targets for patients with ovarian cancer. We identified 34 peptides derived from these genes in the HLA class I ligandome of OVCA patient samples as well as various tumor cell lines. For nine peptides we identified potent T-cell clones in the allo-HLA T-cell repertoire of healthy donors, demonstrating these peptides can be recognized by T cells. We made a final selection of four potent and specific TCRs recognizing PRAME or CTCFL peptides presented in different HLA alleles. The three PRAME TCRs, recognizing peptides in HLA-A*02:01, -A*24:02 or -B*07:01, are an essential addition to the currently used TCRs. We demonstrated that these PRAME TCRs exhibit potent antitumor reactivity *in vitro* and *in vivo*. The CTCFL TCR recognizing an HLA-A*02:01 restricted peptide is, to our knowledge, the first CTCFL TCR described to date. The CTCFL TCR-T cells efficiently recognized primary patient-derived OVCA cells, and OVCA cell lines treated with epigenetically regulator DAC. Overall, the four TCRs are considered promising candidates for TCR gene transfer strategies in patients suffering from ovarian cancer or other *PRAME* or *CTCFL* expressing cancers.

We aimed to identify strictly tumor-specific TAAs in ovarian cancer by only selecting DE genes with a FC ≥ 20 compared to all healthy tissues of risk. Not all antigens currently targeted in clinical studies with ovarian cancer patients fulfilled these strict criteria. CAR-T cells targeting extracellular proteins CLDN6, mucin16, mesothelin, folate receptor-α and HER2, are currently investigated in ovarian cancer patients (43). The DE fold change values calculated in our analysis were respectively 137, 12, 6, 3 and 1 (**Supplementary table 2**). According to our DE criteria (FC ≥ 20), we consider CLDN6 a strictly tumor-specific target for ovarian cancer patients. For the other targets the difference between expression in OVCA patient samples and some of the healthy tissues was lower, suggesting possible on-target off-tumor toxicity risks and a narrow therapeutic window (44). Moreover, we question whether the frequently studied TCR targets NY-ESO-1 and MAGE-A4 are optimal targets for the majority of ovarian cancer patients, since the mean expression levels were low in the included TCGA OVCA samples (mean read count ¾ 100).

Currently three clinical studies targeting CLDN6 are ongoing in ovarian cancer patients: a CLDN6 CAR (NCT04503278 (45)), CLDN6 bispecific T cell engager (NCT05317078 (46)) and CLDN6 CAR-NK (NCT05410717). In our study, thus far only T cells reactive against CLDN6 peptide-loaded cells, but not against CLDN6 transduced cells were identified. We, however, anticipate that the three identified CLDN6 peptides can be used for identification of more potent CLDN6-reactive TCRs in the future. To our knowledge these are the first validated CLDN6 peptides found in the HLA ligandome. In general, the number of unique CLDN6-derived peptides will be limited due to shared homology with ubiquitously expressed Claudin-family members. This also counts for CTCFL which has homology with its ubiquitously expressed paralog CTCF. Based on serious side effects in patients treated with a TCR targeting MAGE-A3 and -A9, that was cross-reactive with MAGE-A12 expressed in brain (47), overlap or minor differences in peptide sequences between tumor and ubiquitously expressed antigens is probably not acceptable. Recently two TCRs targeting CLDN6 peptides that were predicted to bind to HLA-A*02:01 or HLA-DR*04:04 have been identified (15). Considering the shared homology of the HLA-A*02:01 binding peptide with CLDN9, the safety of this TCR has to be carefully evaluated.

The three identified PRAME TCRs demonstrated potent and specific antitumor reactivity *in vitro* and *in vivo* and pose a valuable addition to the currently used TCRs targeting the SLL or VLD peptide presented in HLA-A*02:01. Only TCR 16.3C1^PRAME/LYV/A24^ showed HLA cross-reactivity against the globally infrequent alleles HLA-B*37:01 (3.23%) and HLA-B*38:01 (1.72%) (40) (**Figure 3C**), implicating this TCR is not suitable for the group of patients expressing these HLA alleles. Furthermore, clone 8.10C4^PRAME/SPS/B7^ demonstrated some reactivity against *PRAME* negative imDCs. However, given the lack of reactivity by the TCR-T cells towards imDCs, we hypothesize the reactivity is a result of non-TCR mediated recognition, for example induced by a killer immunoglobulin-like receptor expressed on the T-cell clone. Given the broad and high *PRAME* expression in many tumor types (**Supplementary Figure 2**), we expect the PRAME TCRs to be valuable for treatment of other *PRAME* positive tumors as well. PRAME-reactive TCRs are currently investigated in a variety of tumor types: myeloid and lymphoid neoplasms (NCT03503968), acute myeloid leukemia, myelodysplastic syndrome and uveal melanoma (NCT02743611), and various solid tumors including ovarian cancer (NCT03686124 (48) and a TCR/anti-CD3 bispecific fusion protein in NCT04262466 (49)). Especially for PRAME our strategy to isolate high-avidity T cells in the allo-HLA T-cell repertoire was essential, since low *PRAME* expression in mDCs (3.2%) and PTECs (1.3%) (**Supplementary Figure 6**) implicate self-tolerance to PRAME in the autologous T-cell repertoire. Previously, we indeed demonstrated that PRAME-specific T-cell clones derived from the autologous T-cell repertoire lacked reactivity against endogenously processed PRAME and showed lower peptide sensitivity compared with T-cell clones derived from the allo-HLA T-cell repertoire (39). Apart from the T-cell repertoire, selecting the accurate peptide is crucial for clinical efficacy of TCR-based therapy as well. We identified 23 naturally expressed PRAME peptides, of which 8 peptides were presented in HLA-A*02:01. We were not able to identify the often used VLD peptide presented in HLA-A*02:01, which may suggest this peptide is not optimally processed and presented in PRAME positive tumor cells.

Although CTCFL has been proposed as an attractive tumor target given the restricted expression profile and several oncogenic properties, studies investigating CTCFL-targeting therapies are still limited. CTCFL, also named brother of the regulator of imprinted sites (BORIS), is a DNA binding protein and plays a central role in gene regulation by acting as a transcription factor of testis-specific genes, including some CTAs (50). By interfering with cellular processes such as apoptosis, proliferation and immortalization, CTCFL exhibits several oncogenic properties (50). In ovarian cancer *CTCFL* expression indeed correlates with advanced stage and decreased survival (51). In other tumor types *CTCFL* expression has also been detected, although expression data have been contradictory (52). According to the TCGA data, *CTCFL* is mainly expressed in ovarian cancer (**Supplementary Figure 2**). We also demonstrated high *CTCFL* expression in most primary OVCA patient samples, and demonstrated reactivity of the CTCFL TCR-T cells against the primary patient-derived OVCA cells of an HLA-A*02:01 positive OVCA patient. With the exception of the cervical cancer cell line Ca Ski, no expression was observed in OVCA tumor cell lines (**Figure 2A**). Since *CTCFL* expression is epigenetically regulated, treatment with demethylating agent DAC has previously been shown to upregulate *CTCFL* in OVCA cell lines (42). We also observed increased expression of *CTCFL*, leading to increased reactivity by the CTCFL TCR-T cells against DAC-treated OVCA cell lines (**Figures 6E, G**). We also demonstrated this for the HSS3^PRAME/SLL/A2^ TCR-T cells (**Supplementary Figure 9**), which is in line with previous findings using PRAME-reactive T cells and DAC-treated leukemic cell lines (20). These preclinical findings demonstrate that pre-treatment with DAC may increase reactivity of transferred TCR-T cells in patients. However, clinical data on effectivity or potential toxicity risks, if DAC upregulates gene expression also in non-malignant cells, is limited.

In summary, we present a selection of strictly and highly expressed DE genes in ovarian tumors, combined with a set of naturally expressed peptides. We expect this selection to broaden the applicability of T-cell therapies in patients with ovarian cancer. In addition, we consider the three PRAME TCRs (DSK3^PRAME/QLL/A2^, 16.3C1^PRAME/LYV/A24^ and 8.10C4^PRAME/SPS/B7^) and CTCFL TCR (39.2E12^CTCFL/KLH/A2^) to be promising candidates for the treatment of patients with ovarian cancer, and also for other *PRAME* or *CTCFL* expressing cancers.

## Data availability statement

The mass spectrometry proteomics data have been deposited to the ProteomeXchange Consortium *via* the PRIDE (53) partner repository with the dataset identifier PXD040651.

## Ethics statement

The studies involving human participants were reviewed and approved by Institutional Review Board of the LUMC (approval number 3.4205/010/FB/jr) and the METC-LDD (approval number HEM 008/SH/sh), for samples of LUMC Biobank for Hematological Diseases. For the OVCA samples this was approved by the Institutional Review Board of the LUMC (approval number L18.012) and Central Committee on Research Involving Human Subjects (approval number NL63434.000.17). The patients/participants provided their written informed consent to participate in this study. The animal study was reviewed and approved by National Ethical Committee for Animal Research (AVD116002017891).

## Author contributions

RA designed, performed, analyzed, and interpreted all experiments and wrote the manuscript. ST performed the differential gene expression analysis. AW, MM and SS performed *in vitro* experiments. MHM and TW performed *in vivo* experiments. DR performed qPCR and constructed retroviral expression vectors. RH determined TRAV and TRBV usage and constructed retroviral expression vectors. DS generated and analyzed peptide elution data and produced pMHC-multimers. AR performed and analyzed mass spectrometry experiments. EV provided ovarian cancer patient samples and cell lines. PV produced and analyzed MS data. JF supervised the study and revised the manuscript. MH designed and interpreted the experiments, conceptualized and supervised the study, and revised the manuscript. All authors contributed to the article and approved the submitted version.
