# Supplementary material for: PRAME and CTCFL-reactive TCRs for the treatment of ovarian cancer
Source: Front Immunol. 2023 Mar 21;14:1121973. doi: 10.3389/fimmu.2023.1121973 (PMC10070997; doi:10.3389/fimmu.2023.1121973)
Supplement: Supplementary file 1 [file DataSheet_1.pdf]

Supplemental Figures and Tables

PRAME and CTCFL-reactive TCRs for the treatment of ovarian cancer

Rosa A. van Amerongen[1], Sander Tuit[1] , Anne K. Wouters[1], Marian van de Meent[1], Sterre L. Siekman[1], Miranda H. Meeuwsen[1], Tassilo L. A. Wachsmann[1], Dennis F.G. Remst[1], Renate S. Hagedoorn[1], Dirk M. van der Steen[1], Arnoud H. de Ru[2], Els M.E. Verdegaal[3], Peter A. van Veelen[2], J.H. Frederik Falkenburg[1], and Mirjam H.M. Heemskerk[1].

1 Department of Hematology, Leiden University Medical Center, Leiden, Netherlands;

2 Center for Proteomics and Metabolomics, Leiden University Medical Center, Leiden, Netherlands;

3 Department of Medical Oncology, Oncode Institute, Leiden University Medical Center, Leiden, Netherlands.

Supplemental figure 1. *PRAME*, *CTCFL* and *CLDN6* expression in OVCA and healthy tissues

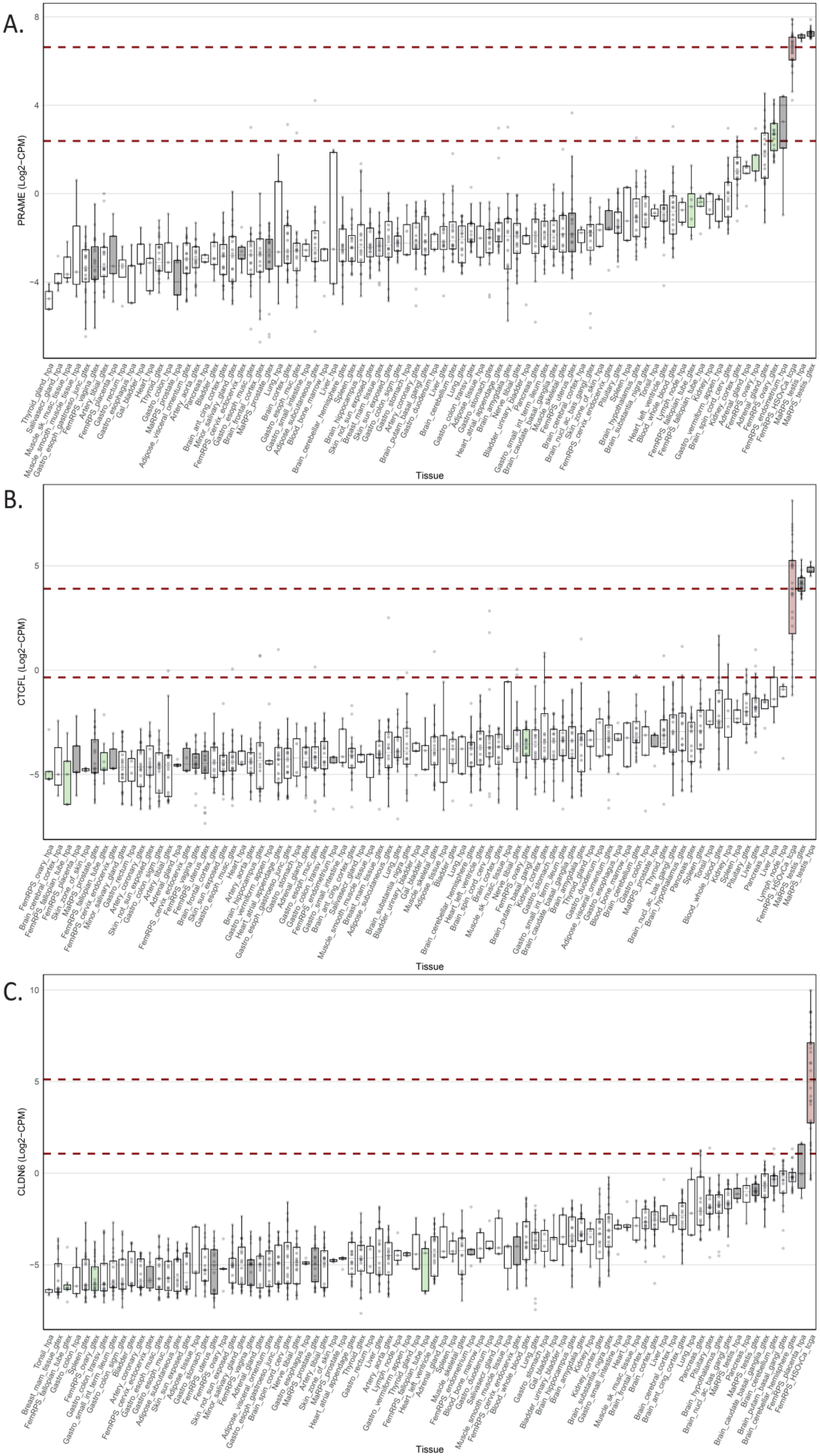

Supplemental figure 2. *PRAME*, *CTCFL* and *CLDN6* expression in tumor samples

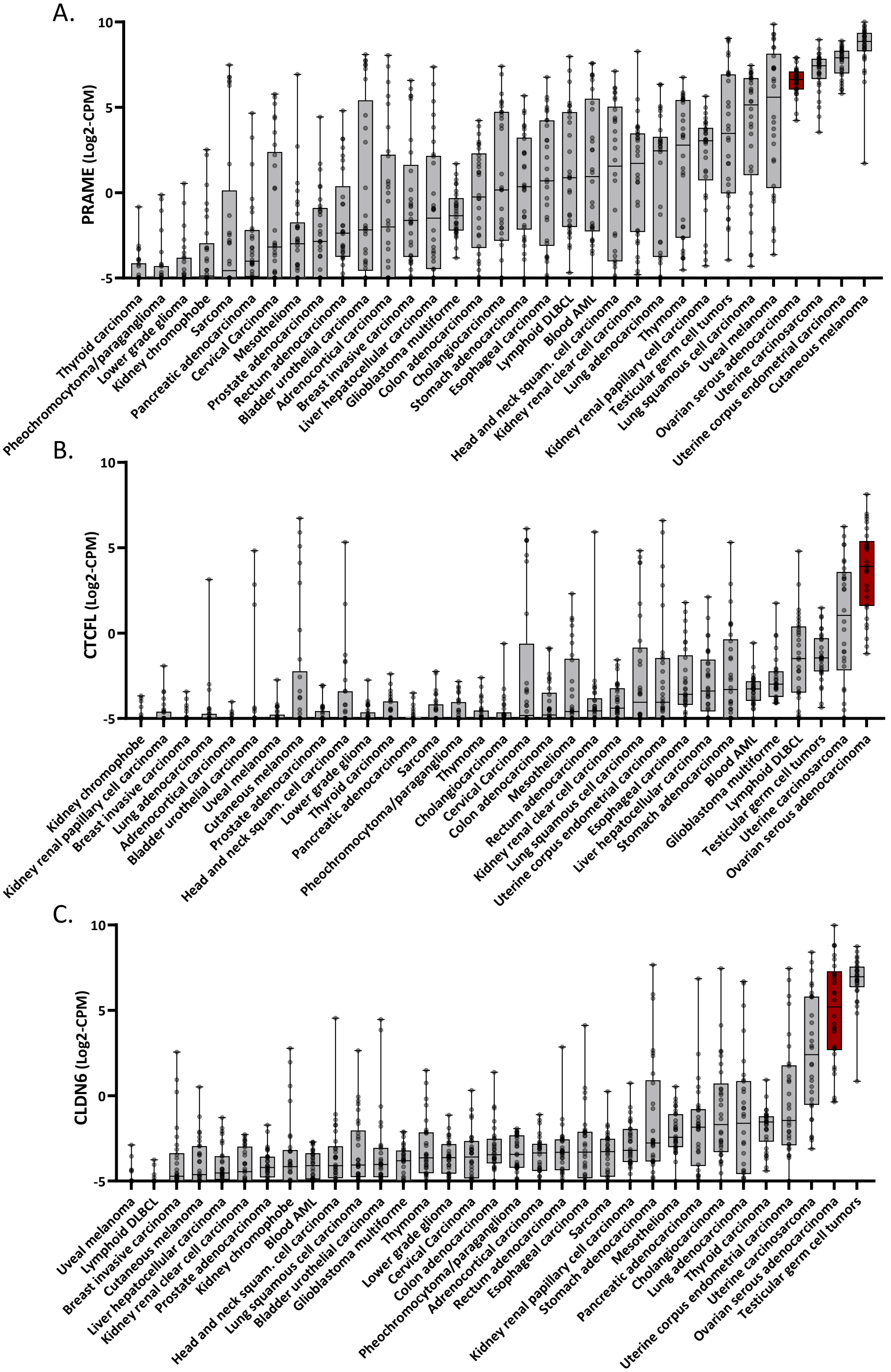

Supplemental figure 3. Examples of mass spectra comparisons of eluted (top) and synthetic (bottom) peptides

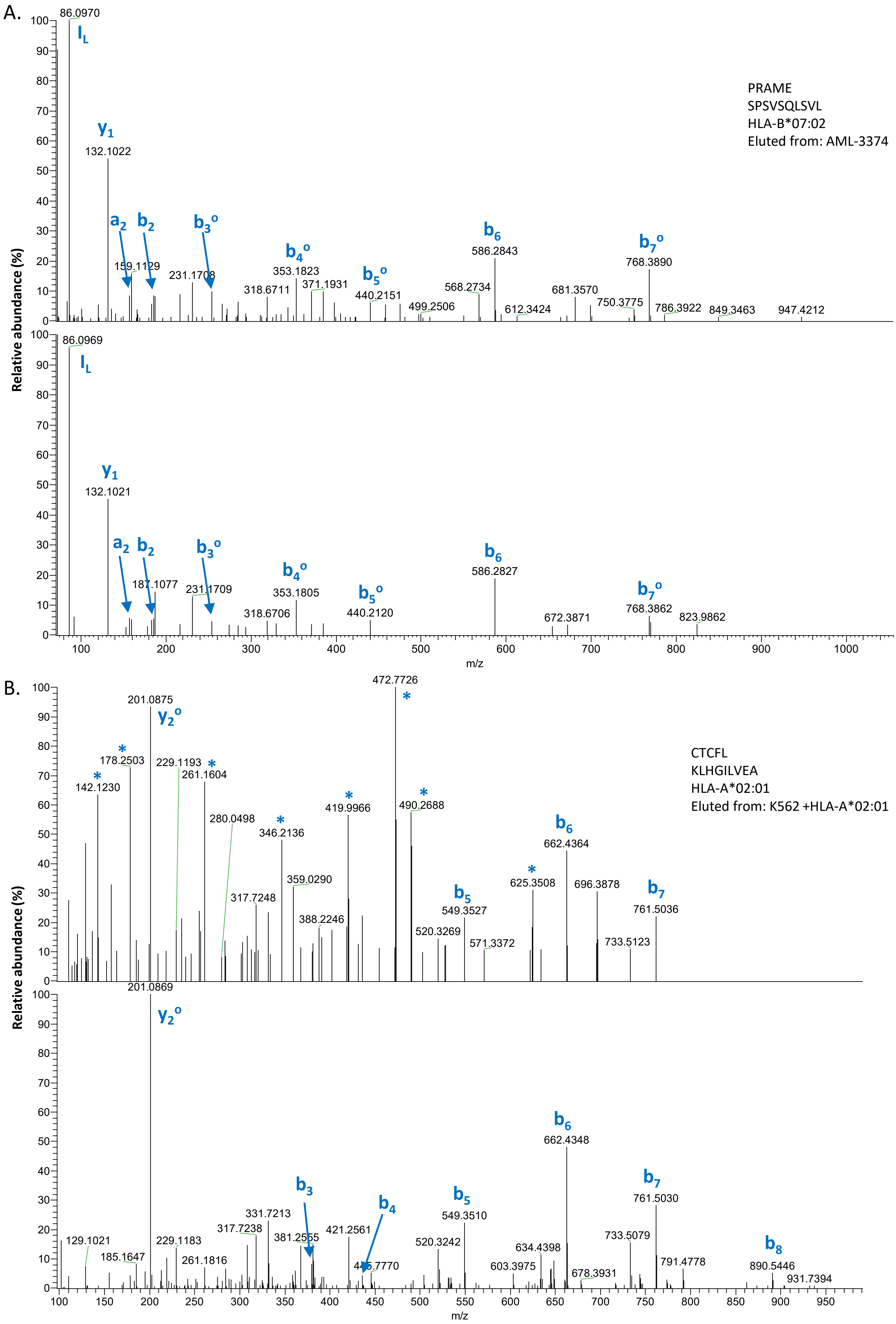

Supplemental figure 3. Examples of mass spectra comparisons of eluted (top) and synthetic (bottom) peptides

C.

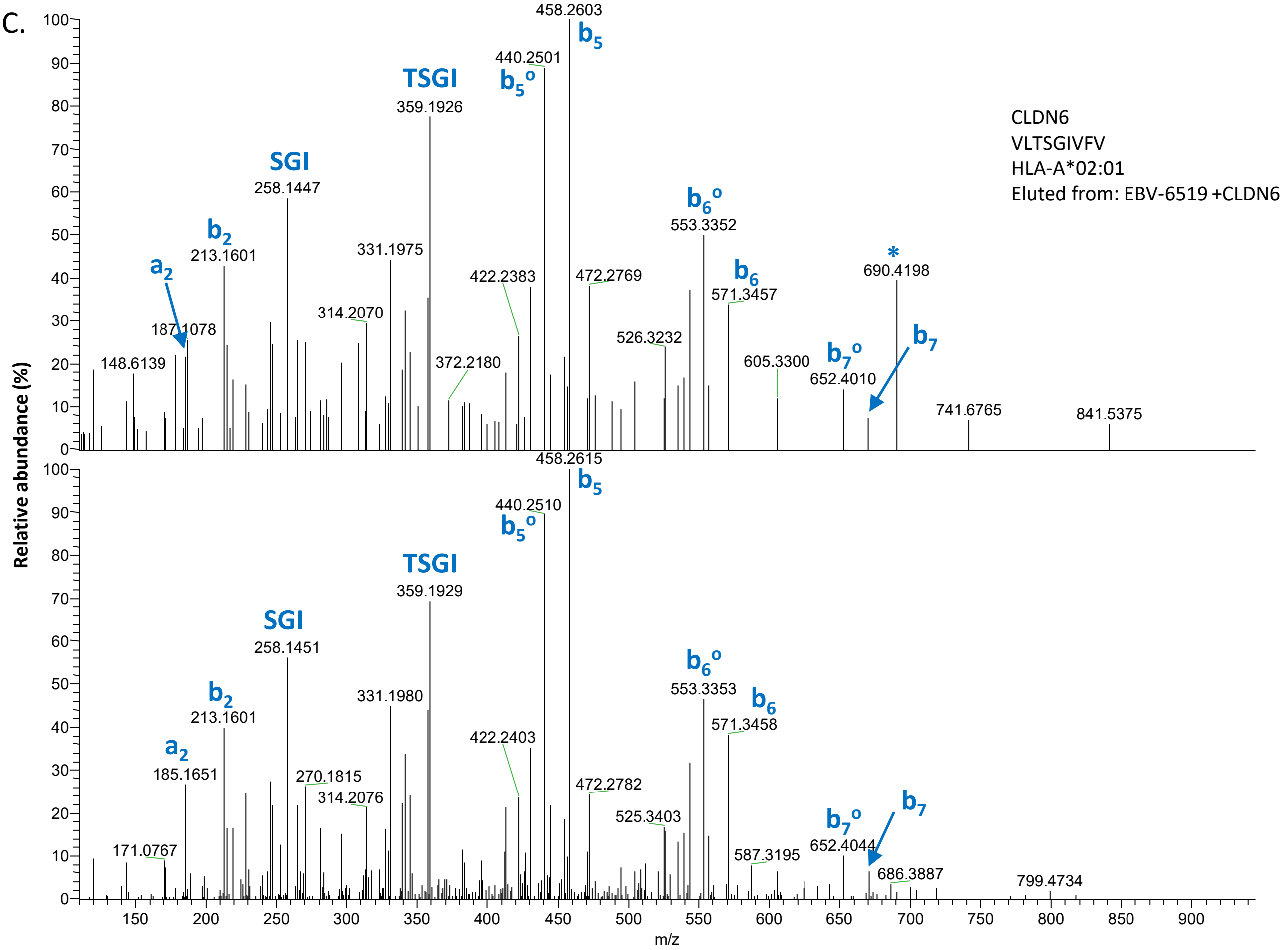

Supplemental figure 4. Location of peptides and used qPCR primers in aligned CTCFL variants

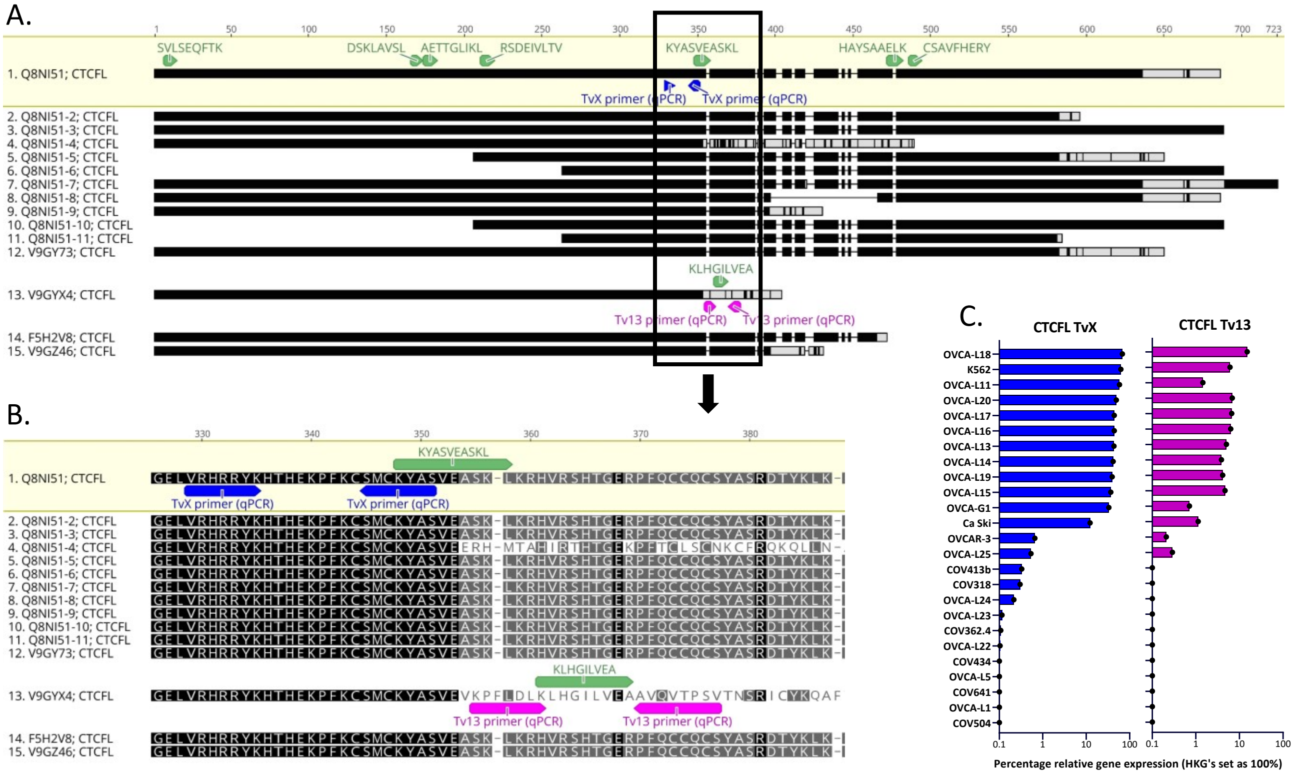

Supplemental figure 5. Peptide-specificity and target gene recognition summarized for the selected T-cell clones

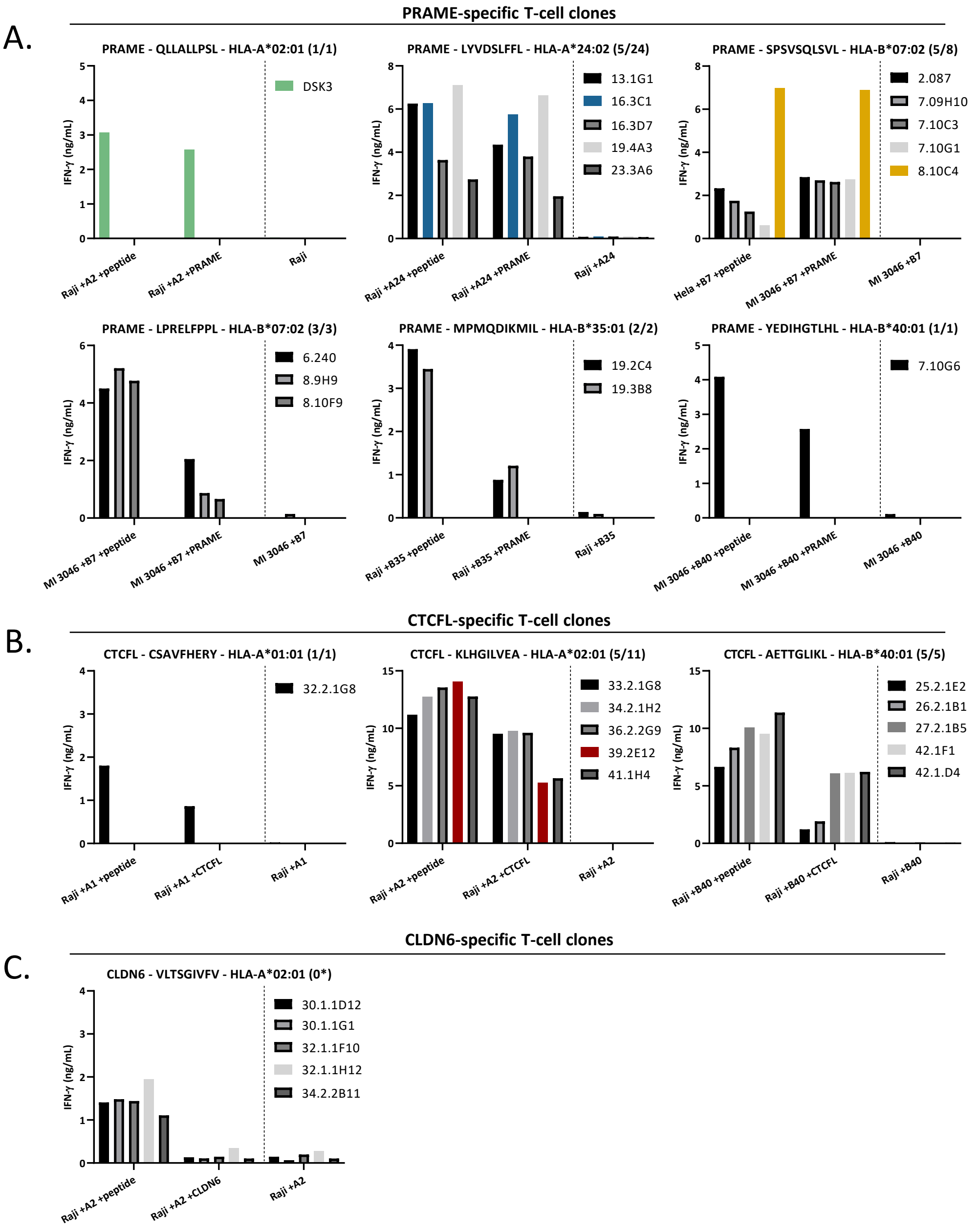

Supplemental figure 6. *PRAME*, *CTCFL* and *CLDN6* expression in primary patient-derived OVCA samples and healthy cell subsets (qPCR)

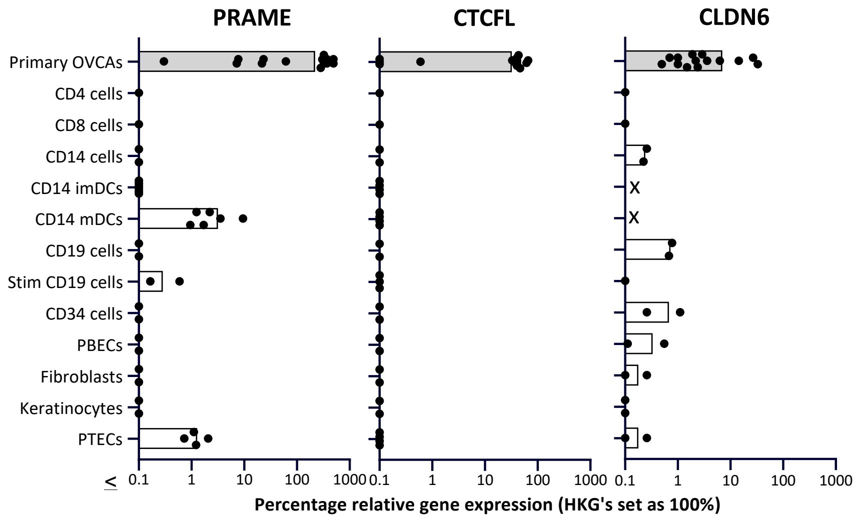

Supplemental figure 7. Recognition and killing of peptide-loaded tumor cell lines by the PRAME TCR-T cells

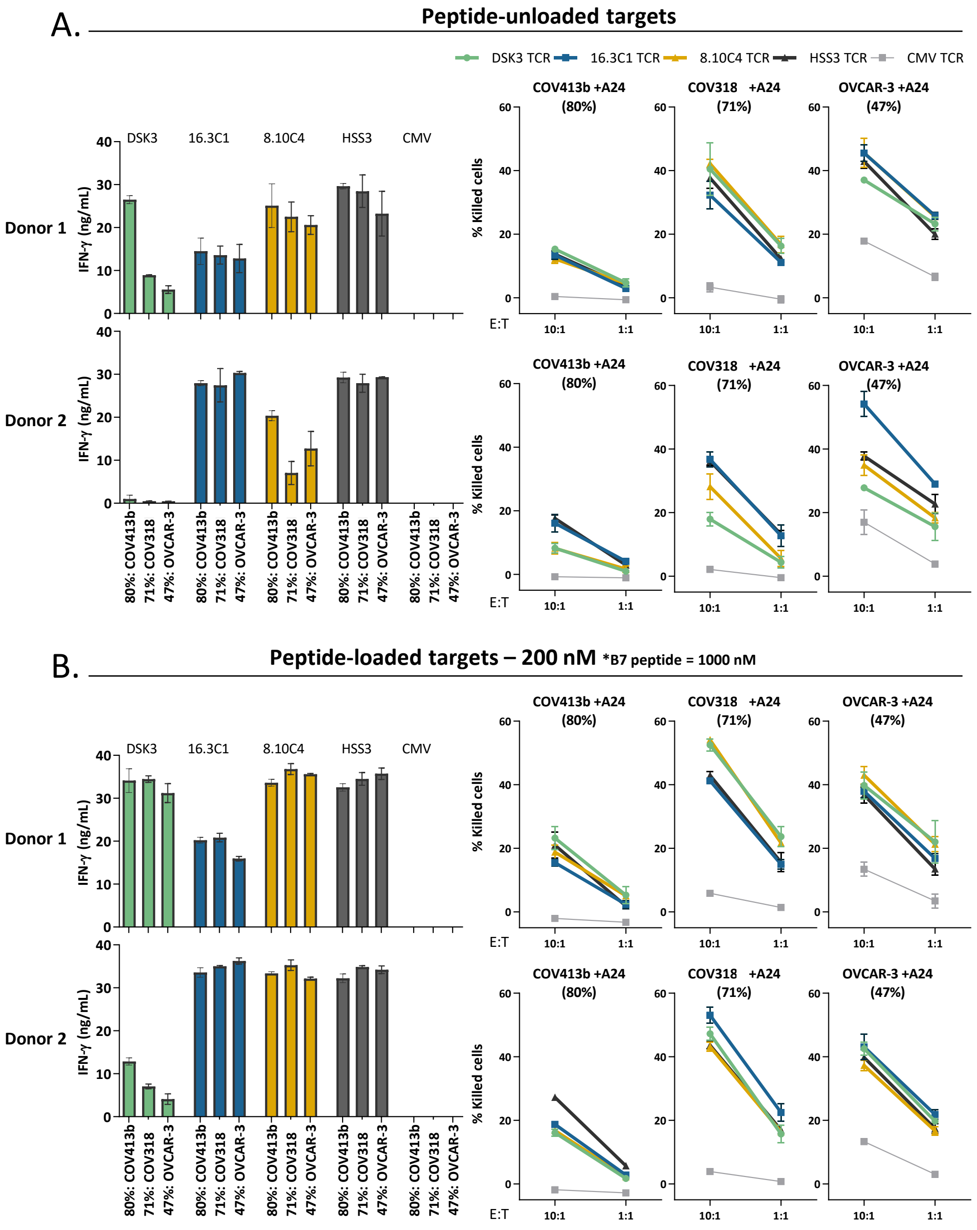

Supplemental figure 8. Recognition and killing of peptide-loaded tumor cell lines by the CTCFL TCR-T cells

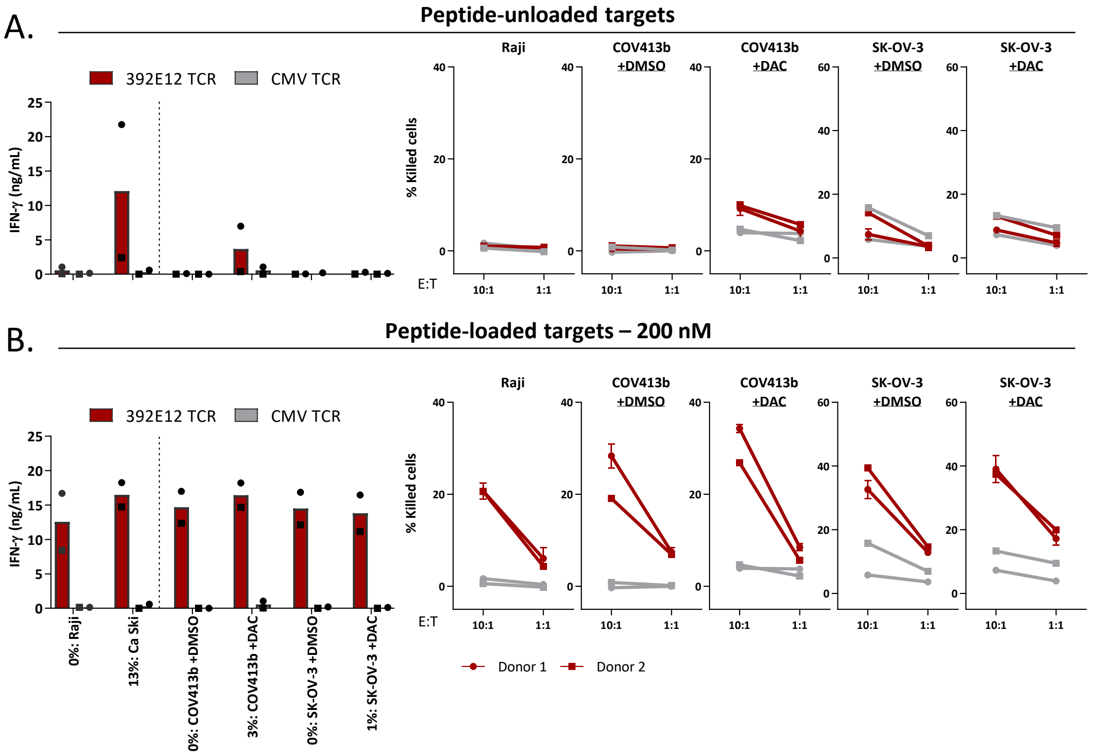

Supplemental figure 9. Increased recognition and killing of DAC treated OVCA cells by PRAME TCR-T cells

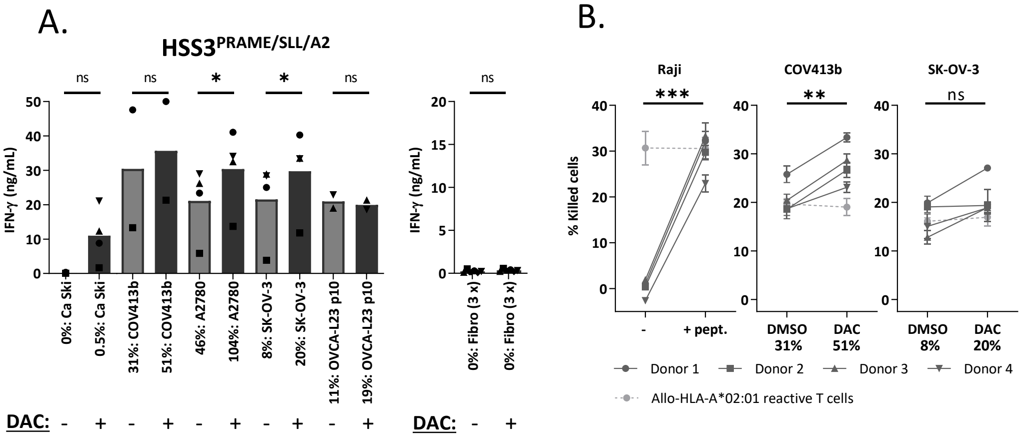

Legends – Supplemental Figures

Supplemental figure 1. *PRAME*, *CTCFL* and *CLDN6* expression in OVCA and healthy tissues

Boxplots depicting (**A**) *PRAME*, (**B**) *CTCFL*, and (**C**) *CLDN6* expression in ovarian cancer (TCGA data, n=30) and across 51 (GTEx data, n=6-20) and 32 (HPA data, n=3-5) healthy tissue types, respectively. Green, red, grey and white boxplots represent healthy ovary and fallopian tube, ovarian tumor, healthy reproductive tissues and all remaining healthy tissues, respectively. Boxplots extend from first to third quartile, the horizontal line represent the median expression value. The whiskers represent the minimum and maximum expression value (1.5 IQR from the first and third quartile). Outliers are defined as being 1.5*IQR or more above the third or below the first quartile, respectively. The upper and lower red dashed lines represent the median expression value and the 20 times lower expression value, respectively. X-axis abbreviations are described in supplement table 1. (FC: fold change, GTEx: genotype-tissue expression, HPA: human protein atlas, IQR: interquartile range, log2-CPM: log2-transformed counts per million, TCGA: The cancer genome atlas)

Supplemental figure 2. *PRAME*, *CTCFL* and *CLDN6* expression in tumor samples

Boxplots depicting (**A**) PRAME, (**B**) CTCFL, and (**C**) CLDN6 expression across 33 different tumor types (TCGA data, n=30). Red and grey boxplots represent ovarian tumor and all other tumor types, respectively. Boxplots extend from first to third quartile, where the horizontal line represent the median expression value. The whiskers represent the minimum and maximum expression value (1.5 IQR from the first and third quartile). Outliers are defined as being 1.5*IQR or more above the third or below the first quartile, respectively. X-axis abbreviations are described in supplement table 1. (IQR: interquartile range, log2-CPM: log2-transformed counts per million, TCGA: The cancer genome atlas)

Supplemental figure 3. Examples of mass spectra comparisons of eluted (top) and synthetic (bottom) peptides

(**A**) Tandem mass spectra comparison of peptide SPSVSQLSVL derived from PRAME presented in HLA-B*07:02, (**B**) peptide KLHGILVEA derived from CTCFL presented in HLA-A*02:01, and (**C**) peptide VLTSGIVFV derived from CLDN6 presented in HLA-A*02:01. In all mass spectra sequence specific fragment ions have been indicated. Superscript open circles denote water loss. Internal fragment ions are indicated by their sequence. Interfering fragment ions, caused by co-isolation of the precursor ions and do not belong to the indicated peptide sequence, are marked with an asterisk. The interference is particularly high for peptide KLHGILVEA, which was identified at a very low abundance.

Supplemental figure 4. Location of peptides and used primers in aligned CTCFL variants

(**A**) Protein sequence alignment of the 15 protein variants derived from the CTCFL gene isoforms due to alternative splicing, according to the UniProt database.[31] The canonical sequence is selected as reference sequence, differences with the reference sequence are depicted in white. Shown are the identified CTCFL peptides in green, the primer specific for the canonical sequence (CTCFL TvX) in blue and the primer specific for variant 13 (CTCFL Tv13) in pink. (**B**) Zoom in of the selection marked in (A). (**C**) *CTCFL* expression measured by qPCR using primer TvX and Tv13. Shown is percentage relative expression to the three HKGs *GUSB*, *VPS29* and *PSMB4*, which was set at 100%. (HKGs: housekeeping genes.)

Supplemental figure 5. Peptide-specificity and target gene recognition summarized for the selected T-cell clones

Summary graphs with IFN-γ production (ng/mL) of the isolated T-cell clones recognizing both target cells loaded with peptides (1 µM) and target cells transduced with the target gene. Each graph shows the most potent and specific T-cell clones per specificity for (**A**) six PRAME peptides and (**B**) three CTCFL peptides. The number of T-cell clones shown and the total number isolated are given between brackets. For three specificities only the five most potent T-cell clones are shown. *No T-cell clones recognizing transduced CLDN6 were identified, the five best T-cell clones recognizing peptide-loaded cells are shown in (**C**). In all target cells the HLA allele that presents the targeted peptide is introduced by transduction (+A2, +A24 or +B7). Bars depict averaged duplicate values and the four T-cell clones ultimately selected for TCR transduction are colored.

Legends – Supplemental Figures

Supplemental figure 6. *PRAME*, *CTCFL* and *CLDN6* expression in primary patient-derived OVCA samples and healthy cell subsets (qPCR)

PRAME, CTCFL (TvX) and CLDN6 mRNA gene expression in primary patient-derived OVCA samples and various healthy cell subsets. Expression was measured by qPCR and is shown as percentage relative to the three KKGs *GUSB*, *VPS29* and *PSMB4*, which was set at 100%. (OVCA: primary ovarian cancer patient sample, imDCs and mDCs: immature and mature dendritic cells, HKGs: housekeeping genes, PBECs: primary bronchus epithelial cells, PTECs: proximal tubular epithelial cells.)

Supplemental figure 7. Recognition and killing of peptide-loaded tumor cell lines by the PRAME TCR-T cells

Recognition and killing of unloaded and peptide-loaded OVCA cell lines by the PRAME TCR-T cells and CMV TCR-T cells. Data is shown for two different donors. (**A**) IFN-γ production (ng/mL) of the TCR-T cells cocultured overnight with three OVCA cell lines (E:T = 1:6), showing mean and SD of technical duplicates. Percentage killed cells (E:T = 10:1 and 1:1) measured in a 6-hour ^{51}Cr-release assay, showing mean and SD of technical triplicates. All OVCA cell lines are wildtype A2+ and B7+, and A24 is introduced by transduction. (**B**) IFN-γ production and killing of OVCA cell lines loaded with the QLL/A2 peptide (200 nM), LYV/A24 peptide (200 nM), SLL/A2 peptide (200 nM) and SPS/B7 peptide (1000 nM). Percentage relative PRAME expression is depicted, as determined by qPCR.

Supplemental figure 8. Recognition and killing of peptide-loaded tumor cell lines by the CTCFL TCR-T cells

Recognition and killing of unloaded and peptide-loaded target cells by the CTCFL TCR-T cells and CMV TCR-T cells. Data is shown for two different donors. (**A**) IFN-γ production (ng/mL) of the TCR-T cells cocultured overnight with 1 µM DAC or DMSO treated target cells (E:T = 1:6), bars represent mean and symbols depict averaged technical duplicates from two different donors. And percentage killed cells (E:T = 10:1 and 1:1) measured in a 6-hour ^{51}Cr-release assay, symbols show mean and SD of technical triplicates from two different donors. COV413b is wildtype A2+ and Raji and SK-OV-3 are transduced with A2. (**B**) IFN-γ production and killing of the same target cells loaded with the KLH/A2 peptide (200 nM). Percentage relative CTCFL (TvX) expression is depicted, as determined by qPCR. (DAC: 5-aza-2'-deoxycytidine, E:T: effector:target ratio)

Supplemental figure 9. Increased recognition and killing of DAC-treated OVCA cells by PRAME TCR-T cells

Recognition and killing of DAC-treated target cells by the HSS3$^{PRAME/SLL/A2}$ TCR-T cells, shown for four different donors. (**A**) IFN-γ production (ng/mL) of the PRAME TCR-T cells cocultured overnight with 7 days 1 µM DAC or DMSO treated fibroblasts and tumor cell cells (E:T = 1:6). Bars represent mean and symbols depict averaged duplicate values from four different donors tested in two independent experiments. (**B**) Percentage killed cells (E:T = 10:1 and 1:1) measured in a 6-hour ^{51}Cr-release assay. Mean and SD depict technical triplicates from four different donors tested in two independent experiments, at E:T ratio 10:1. Cytotoxic capacity of an allo-HLA-A*02:01 reactive T-cell clone recognizing HKG USP11 is shown for the different conditions. (**A-B**) Recognition and killing of DMSO and DAC treated cells, or Raji cells loaded with and without peptide, are compared using a paired t-test (two-sided). Percentage relative PRAME expression is depicted, as determined by qPCR. (ns: not significant, DAC: 5-aza-2'-deoxycytidine, E:T: effector:target ratio)

Supplemental table 3. Overview of the materials included in HLA ligandome analyses

	Name	Type	Amount	HLA class 1 typing	PRAME	CTCFL	CLDN6	Eluted OVCA peptides:
A.	OVCA-G1	Primary OVCA	8 gram	A*01:01, A*11:01, B*08:01, B*35:01, C*04:01, C*07:01	7%	33%	14%	x
	OVCA-L1	Primary OVCA	n.d.	A*11:01, A*24:02, B*18:01, B*40:01, C*07:04, C*08:01	3%	0%	1%	PRAME (1x)
	OVCA-L5	Primary OVCA	2 gram	A*01:01, A*31:01, B*08:01, B*35:01, C*07:01, C*15:02	0%	0%	1%	x
	OVCA-L10	Primary OVCA	17 gram	A*02:01, A*11:01, B*07:02, B*55:01, C*03:03, C*07:02	22%	0%	2%	PRAME (1x)
	OVCA-L11	Primary OVCA	5 gram	A*02:01, A*03:01, B*07:02, B*44:02, C*07:02, C*12:03	23%	62%	6%	x
	OVCA-L14	Primary OVCA	3 gram	A*02:01, A*24:02, B*07:02, B*35:03, C*07:02, C*12:03	373%	41%	1%	x
	OVCA-L18	Primary OVCA	20 gram	A*01:01, A*02:01, B*08:01, B*15:01, C*03:04, C*07:01	283%	66%	3%	x
	OVCA-L23	Primary OVCA	$7x10^9$	A*02:01, A*26:01, B*38:01, B*44:02, C*05:01, C*12:03	62%	0%	33%	PRAME (6x)
B.	COV362.4	OVCA cell line	$2x10^9$	A*03:01, B*40:01, C*03:04	18%	0%	1%	CTCFL (1x), PRAME (5x)
	COV413b	OVCA cell line	$4x10^9$	A*02:01, B*07:02, C*07:02	80%	0%	71%	PRAME (11x)
C.	AML-6711	Primary AML	$65x10^9$	A*02:01, A*24:02, B*07:02, B*15:01, C*03:04, C*07:02	18%	nd	nd	PRAME (2x)
	AML-6498	Primary AML	$149x10^9$	A*11:01, A*68:01, B*35:01, B*35:03, C*04:01	15%	nd	nd	PRAME (2x)
	AML-3374	Primary AML	$500x10^9$	A*01:01, A*24:02, B*07:02, B*08:01, C*07:01	nd	nd	nd	PRAME (1x)
	EBV-5098	EBV-LCL	$20x10^9$	A*02:01, B*07:02, C*07:02	9%	nd	nd	PRAME (7x)
	U266	MM cell line	$10x10^9$	A*03:01, A*02:01, B*40:01, B*07:02, C*07:02, C*03:04	4%	0%	nd	PRAME (4x)
D.	EBV-6268 +CLDN6	EBV-LCL	$2x10^9$	A*02:01, A*24:02, B*35:02, B*44:02, C*04:01, C*05:01	x	x	High	x
	EBV-5098 +CLDN6	EBV-LCL	$2x10^9$	A*02:01, B*07:02, C*07:02	x	x	High	x
	EBV-9603 +CLDN6	EBV-LCL	$2x10^9$	A*01:01, A*24:02, B*08:01, B*39:06, C*07:01, C*07:02	x	x	High	CLDN6 (2x)
	EBV-6519 +CLDN6	EBV-LCL	$2x10^9$	A*02:01, A*11:01, B*35:01, B*38:01, C*04:01, C*12:03	x	x	High	CDLN6 (1x)
	K562 +A1, +A2, +A3, +A24, +B7, +B8	CML cell line	(6 x) $2x10^9$	x	230%	62%	3%	CTCFL (6x), PRAME (2x)
	K562 +CTCFL TvX +A2 +A24	CML cell line	$2x10^9$	x	230%	62%	3%	CTCFL (1x)
	TMD8 +A2, +B7	DLBCL cell line	(2 x) $2x10^9$	A*02:07, B*15:01, B*46:01, C* 01:02	76%	0%	1%	PRAME (9x)

Supplemental table 4. Identified OVCA gene-derived HLA class I peptides

	Gene	Peptide	HLA	Sample / cell line source	BMI
1	PRAME	QLLALLPSL	A*02:01	TMD8 +A2, EBV-5098	37
2	PRAME	SLLQHLIGL	A*02:01	**COV413b**, U266, TMD8 +A2, AML-6711, EBV-5098	54
3	PRAME	ALLERASATL	A*02:01	**OVCA-L23, COV413b**	51
4	PRAME	ALQSLLQHL	A*02:01	**OVCA-L23, COV413b**	26
5	PRAME	GLSNLTHVL	A*02:01	**OVCA-L23, COV413b**	25
6	PRAME	QLDSIEDLEV	A*02:01	**OVCA-L23**	46
7	PRAME	RLDQLLRHV	A*02:01	**COV413b**, TMD8 +A2	45
8	PRAME	VQLDSIEDLEV	A*02:01	**OVCA-L23**, TMD8 +A2, EBV-5098	76
9	PRAME	RLVELAGQSLLK	A*03:01	**COV362.4**	25
10	PRAME	SPRRLVELAGQSL	B*07:02	**COV413b**, AML-6711, TMD8 +B7, EBV-5098	30
11	PRAME	MPMQDIKMIL	B*07:02	TMD8 +B7, AML-6498	25
12	PRAME	SPSVSQLSVL	B*07:02	**COV413b**, EBV-5098, TMD8 +B7, AML-3374, U266	65
13	PRAME	LPRELFPPL	B*07:02	EBV-5098, K562+B7	26
14	PRAME	LPSLSHcSQL	B*07:02	**COV413b**	29
15	PRAME	RPSmVWLSA	B*07:02	**COV413b**	31
16	PRAME	SPYLGQMINL	B*07:02	**COV413b**, TMD8+B7	54
17	PRAME	DEALAIAAL	B*18:01/40:01/44:02	**OVCA-L1, OVCA-L23, COV413b**	42
18	PRAME	MPMQDIKMIL	B*35:01	TMD8 +B7, AML-6498	25
19	PRAME	LPRELFPPL	B*35:01	EBV-5098, K562+B7	26
20	PRAME	YEDIHGTLHL	B*40:01	**COV362.4**, U266	42
21	PRAME	FDGRHSQTL	B*40:01	**COV362.4**	39
22	PRAME	VELAGQSLL	B*40:01	**COV362.4**	45
23	PRAME	AAFDGRHSQTL	C*03:03/04	**OVCA-L10, COV362.4**, U266	43
24	CTCFL	CSAVFHERY	A*01:01	K562+A1	43
25	CTCFL	RSDEIVLTV	A*01:01	K562+A1	37
26	CTCFL	KLHGILVEA	A*02:01	K562+A2	12
27	CTCFL	HAYSAAELK	A*03:01	K562+A3	56
28	CTCFL	SVLSEQFTK	A*03:01	K562+A3	57
29	CTCFL	KYASVEASKL	A*24:02	K562+CTCFL+A2+A24	64
30	CTCFL	DSKLAVSL	B*08:01	K562+B8	35
31	CTCFL	AETTGLIKL	B*40:01	**COV362.4**	51
32	CLDN6	GPSEYPTKNYV	A*01:01	EBV-9603 +CLDN6	25
33	CLDN6	VLTSGIVFV	A*02:01	EBV-6519 +CLDN6	23
34	CLDN6	DSKARLVL	B*08:01	EBV-9603 +CLDN6	37

Legends – Supplemental Tables

Supplemental table 1. Samples included in the differential gene expression analysis

Dataset, annotations and donor characteristics of the 2202 samples included in the differential gene expression analysis.

Supplemental table 2. Minimal fold change of all genes in ovarian cancer

The minimal fold change values in ovarian cancer (TCGA data) compared to all healthy tissues at risk (HPA and GTEx data), shown for all 16855 included genes. Genes were defined to be DE when they exhibited a fold change of ≥ 20 (logFC of ≥ 4.32) and FDR adjusted p-value of ≤ 0.05. Gene type according to the Ensembl database. (DE: differentially expressed, FDR: false discovery rate)

Supplemental table 3. Overview of the materials included in HLA ligandome analyses

Primary patient-derived OVCA samples (**A**), OVCA cell lines (**B**), various cell lines and patient samples (**C**), and cells transduced with CTCFL, CLDN6 and/or HLA class I molecules (**D**). Shown are for each material the name, cell type, amount used for peptide elution (gram or number of cells in 10^9), HLA class I typing, relative expression of the target genes as determined by qPCR and number of peptides that are eluted and validated. (OVCA: ovarian carcinoma, AML: acute myeloid leukemia, EBV-LCL: Epstein-Barr virus transformed lymphoblastoid cell lines, MM: multiple myeloma, CML: chronic myeloid leukemia, DLBCL: diffuse large B-cell lymphoma, nd: not determined)

Supplemental table 4. Identified OVCA gene-derived HLA class I peptides

Overview of the 34 OVCA gene-derived peptides identified in the HLA ligandome analyses. The gene, peptide, HLA binding restriction, sample/cell line source, and BMI are listed. Details of the samples and cell lines are listed in Supplemental table 1. (AML: primary acute myeloid leukemia sample, BMI: best Mascot ion score, OVCA: primary ovarian cancer sample)
