# Supplementary material for: PRAME and CTCFL-reactive TCRs for the treatment of ovarian cancer
Source: Front Immunol. 2023 Mar 21;14:1121973. doi: 10.3389/fimmu.2023.1121973 (PMC10070997; doi:10.3389/fimmu.2023.1121973)
Supplement: Supplementary file 2 [file DataSheet_2.pdf]

Supplemental Methods

PRAME and CTCFL-reactive TCRs for the treatment of ovarian cancer

Rosa A. van Amerongen[1], Sander Tuit[1] , Anne K. Wouters[1], Marian van de Meent[1], Sterre L. Siekman[1], Miranda H. Meeuwsen[1], Tassilo L. A. Wachsmann[1], Dennis F.G. Remst[1], Renate S. Hagedoorn[1], Dirk M. van der Steen[1], Arnoud H. de Ru[2], Els M.E. Verdegaal[3], Peter A. van Veelen[2], J.H. Frederik Falkenburg[1], and Mirjam H.M. Heemskerk[1].

1 Department of Hematology, Leiden University Medical Center, Leiden, Netherlands;

2 Center for Proteomics and Metabolomics, Leiden University Medical Center, Leiden, Netherlands;

3 Department of Medical Oncology, Oncode Institute, Leiden University Medical Center, Leiden, Netherlands.

Dissociation primary OVCA patient samples

Solid OVCA patient samples were sliced into small pieces and dead, clotted or non-tumor material was removed. The small tumor-pieces were added to a C-tube (Miltenyi Biotec) with ice cold buffer without detergent and cOmplete Protease Inhibitor (Sigma-Aldrich), to prevent protein degradation. Using a gentleMACS (Miltenyi Biotec) procedure small tumor-pieces were dissociated until an almost homogenous cell solution. Benzonase (Merck) was added in a concentration of 125 IU/mL to remove DNA/RNA complexes during lysis.

Gene expression by Quantitative Polymerase Chain Reaction

Gene expression was quantified by Quantitative Polymerase Chain Reaction (qPCR). Total RNA was isolated using the RNAqueous-Micro Kit (Ambion) or ReliaPrep RNA Cell Miniprep System (Promega). First strand cDNA synthesis was performed with Moloney murine leukemia virus reverse transcriptase and Oligo (dT) primers (Invitrogen by Thermo Fisher Scientific). qPCR was performed using Fast Start TaqDNA Polymerase (Roche) and EvaGreen (Biotium), and gene expression was measured on the Lightcycler 480 (Roche). All samples and genes were run in triplicate with 10 ng cDNA per reaction. Expression was calculated as percentage relative to the average of housekeeping genes GUSB, VPS29 and PSMB4, which was set at 100%. The following primers were used: PRAME (forward: GTTGCTCAGGCACGTGAT, reverse: CCCACTTAGACTCAGGACACTTA), CTCFL (TvX) (forward: GTCCGACACAGGCGCTATAA, reverse: CCACACTGGCATACTTGCAC), CTCFL variant 13 (Tv13) (forward: AAAGCCATTCTTGGACTTGAAGC, reverse: TACACTTGGAGTAACTTGTACAGCA), CLDN6 (forward: CAGGGGTCCTGACGCTAATC, reverse: AGCCACCAGGGGGTTATAGA), GUSB (forward: ACTGAACAGTCACCGACGAG, reverse: GGAACGCTGCACTTTTTGGT), PSMB4 (forward: GTTTCCGCAACATCTCTCGC, reverse: CATCAATCACCATCTGGCCG), VPS29 (forward: TGAGAGGAGACTTCGATGAGAATC, reverse: TCTGCAACAGGGCTAAGCTG).

HLA class I-peptide elution procedure, fractionation and mass spectrometry

Peptide elution was performed as outlined previously.[1,2] In short, the cell pellets were lysed and subjected to an immunoaffinity column to collect bound peptide-HLA complexes, with either an HLA class-I antibody (W6/32, ATCC) or an HLA-A*02:01 antibody (BB7.2, ATCC). To separate the peptides, bound peptide-HLA complexes were dissociated with 10% acetic acid and filtrated using a 10 kDa membrane. Eluted peptide pools were either fractionated by strong cation exchange chromatography (SCX)[1] or by high pH reversed phase fractionation (High pH-RP)[3]. SCX and high pH-RP peptide fractions were lyophilized, dissolved in

95/3/0.1 water/ acetonitrile/formic acid v/v/v and subsequently analysed by data-dependent MS/MS on either an LTQ FT Ultra equipped with a nanoflow liquid chromatography 1100 HPLC system (Agilent Technologies) or a Q Exactive mass spectrometer equipped with an easy-nLC 1000 (Thermo Fisher Scientific). Proteome Discoverer version 2.1 (Thermo Fisher Scientific) was used for peptide and protein identification, using the mascot search node for identification (mascot version 2.2.04) and the UniProt Homo Sapiens database (UP000005640; Jan 2015; 67,911 entries). All unique PRAME, CTCFL and CLDN6-derived peptides with a length between 8 and 14 amino acids, preferably a minimal Best Mascot Ion (BMI) score of 20, a mass accuracy of 10 ppm and predicted to bind to a common HLA molecule according to the netMHC peptide binding algorithm[4] were selected as candidate for peptide synthesis and validation.

Peptide synthesis and pMHC-multimer production

In total 34 synthetic peptides were in-house synthesized using standard Fmoc chemistry. By mass spectrometry the tandem mass spectra of the eluted peptides were validated with synthetic peptides. In total 17 pMHC-multimer complexes were generated with minor modifications.[5] In short, monomers consisting of the selected HLA allele heavy chain, human beta-2 microglobulin (B2M) light chain and selected peptide were purified by gel-filtration high-performance liquid chromatography and biotinylated. Subsequently, pMHC-multimers were generated by adding PE-conjugated streptavidin (Invitrogen, Thermo Fisher Scientific).

Cell culture

T cells were cultured in T-cell medium (TCM) composed of Iscove's Modified Dulbecco's Medium (IMDM) (Lonza), 5% heat-inactivated Fetal Bovine Serum (FBS) (Gibco, Thermo Fisher Scientific), 5% human serum (Sanquin Reagents), 1.5% 200 mM L-glutamine (Lonza), 1% 10,000 U/mL penicillin/streptomycin (Pen/Strep; Lonza) and 100 IU/mL IL-2 (Novartis Pharma). Every 10-14 days, $0.2*10^6$ T cells were (re)stimulated with $1*10^6$ irradiated (35 Gy) PBMCs, $0.1*10^6$ irradiated (55 Gy) EBV-LCLs and 0.8 µg/mL phytohemagglutinin (PHA) (Oxoid Microbiology Products, Thermo Fisher Scientific). OVCA cell lines were cultured in Dulbecco's Modified Eagle Medium (DMEM, high glucose 4.5 g/L, NEAA) (Gibco), 8% FBS, 2% 200 mM L-glutamine and 1% 10,000 U/mL Pen/Strep. Other tumor cell lines were cultured in IMDM, 10% FBS, 1.5% L-glutamine and 1% Pen/Strep. Using the PlasmoTest Mycoplasma Detection Kit (InvivoGen), all cell lines were found to be mycoplasma negative. HLA typing was performed and if needed, a single HLA allele was introduced by retroviral transduction. These genes were expressed in MP71 retroviral backbone vectors with marker genes nerve growth factor receptor (NGF-R), green fluorescent protein (GFP), CD34

or mouse CD19 (mCD19). Target cells were enriched for marker gene expression via MACS or FACS and purity was confirmed by flow cytometry. Primary OVCA patient-derived cells were cultured in IMDM, 10% FBS, 1.5% L-glutamine and 1% Pen/Strep, on FBS pre-coated plates.

Antibodies and Flow cytometry

FACS was performed on an LSR II flow cytometer (BD Biosciences) and data was analysed using FlowJo software (TreeStar). T cells were stained with the following conjugated antibodies: CD4 FITC (BD/555346), CD14 FITC (BD/555397), CD19 FITC (BD/555412), CD8 AF700 (Invitrogen/MHCD0829), murine TCR-β (mTCR-β) APC (BD/553174) and pMHC-multimers PE. Target cells transduced with PRAME, CTCFL, CLDN6 or HLA alleles were stained with: NGFR/CD271 APC (Sanbio/CL10013APC), CD34 APC (BD/555824), murine CD19 PE (BD/557399), HLA-A2 PE (BD/558570). Non-malignant hematopoietic subsets with: CD14 FITC (BD/555397), CD19 FITC (BD/555412), CD80 PE (BD/557227) and CD86 PE (BD/555658).

TCR identification and production of retroviral supernatants

TCR α and β chains of the selected T-cell clones were identified by sequencing with minor modifications, as previously described.[6] mRNA was isolated by the Dynabeads mRNA DIRECT Kit (Invitrogen) or total RNA was isolated by the ReliaPrep RNA cell Miniprep System (Promega). TCR cDNA was generated using TCR constant α and β primers, a SA.rt anchor template-switching oligonucleotide (TSO), and SMARTScribe Reverse Transcriptase (Takara, Clontech).[7] The TCR α and β products were generated in a first PCR using Phusion Flash (Thermo Fisher Scientific), followed by a second PCR that was used to include 2-sided barcode sequences for the different T-cell clones. Barcoded TCR PCR products were pooled and TCR sequences were identified by HiSeq or NovaSeq (GenomeScan). The Vα and Vβ families were determined of the NGS data using the MiXCR software and ImMunoGeneTics (IMGT) database.[8] The TCR α (VJ) and β (VDJ) regions were codon optimized, synthesized, and cloned in MP71-TCR-flex retroviral vectors by Baseclear. The MP71-TCR-flex vector already contains codon-optimized and cysteine-modified murine TCR α and β constant domains to optimize TCR expression and increase preferential pairing.[9] Phoenix-AMPHO (ATCC) cells were transiently transfected with the created constructs and after 48 hours retroviral supernatants were harvested and stored at -80°C.

TCR gene transfer to CD8+ T cells

CD8+ T cells were isolated from PBMCs of different donors by MACS using anti-CD8 MicroBeads (Miltenyi Biotech/130-045-201). CD8+ T cells were stimulated with irradiated autologous feeders (40 Gy) and 0.8 µg/mL PHA in 24-well flat-bottom culture plates (Costar). Two days after stimulation, CD8+ T cells were transferred to 24-well flat-bottom suspension culture plates (Greiner Bio-One) for retroviral transduction. These plates were first coated with 30 µg/mL retronectin (Takara, Clontech) and blocked with 2% human serum albumin. Retroviral supernatants were added, and plates were centrifuged at 3000 g for 20 minutes at 4°C. After removal of the retroviral supernatant, $0.3*10^6$ CD8+ T cells were transferred per well. After O/N incubation, CD8+ T cells were transferred to 24-well flat-bottom culture plates (Costar). Seven days after stimulation, CD8+ T cells were MACS enriched for the murine TCR, using mTCR-β APC antibody (BD/553174) and anti-APC MicroBeads (Miltenyi Biotec/130-090-855). Ten days after stimulation, CD8+ T cells were functionally tested and purity was checked by flow cytometry.

^{51}chromium release assay

T-cell mediated cytotoxicity was measured in a 6-hour ^{51}chromium release assay. Target cells were labelled with 100 µCi ^{51}chromium (PerkinElmer) for 1 hour at 37°C, washed, and cocultured with T cells at various E:T ratios in 100 µL TCM per well in 96-well U-bottom culture plates (Costar). Spontaneous and maximum ^{51}Cr release for all targets were measured in separate plates with per well 100 µL TCM or 100 µL TCM with 1% Triton-X 100 (Sigma-Aldrich), respectively. After 6 hours of coculture, 25 µL supernatant was harvested, transferred to 96-well LumaPlates (PerkinElmer) and ^{51}chromium release was measured in counts per minute on a 2450 Microbeta^2 plate counter (PerkinElmer). The percentage of killed target cells was calculated with the following formula = ((experimental release – spontaneous release)/(maximum release – spontaneous release)) *100.

REFERENCES

1. van der Lee DI, Reijmers RM, Honders MW, Hagedoorn RS, de Jong RCM, Kester MGD, et al. Mutated nucleophosmin 1 as immunotherapy target in acute myeloid leukemia. *The Journal of Clinical Investigation.* 2019;129(2):774-785.
2. van Amerongen RA, Hagedoorn RS, Remst DFG, Assendelft DC, van der Steen DM, Wouters AK, et al. WT1-specific TCRs directed against newly identified peptides install antitumor reactivity against acute myeloid leukemia and ovarian carcinoma. *Journal for ImmunoTherapy of Cancer.* 2022;10(6):e004409.

3. de Waard AA, Verkerk T, Hoefakker K, van der Steen DM, Jongsma MLM, Bliss S, et al. Healthy cells functionally present TAP-independent SSR1 peptides: implications for selection of clinically relevant antigens. *bioRxiv.* 2020:2020.2006.2011.146449.
4. Andreatta M, Nielsen M. Gapped sequence alignment using artificial neural networks: application to the MHC class I system. *Bioinformatics.* 2016;32(4):511-517.
5. Burrows SR, Kienzle N, Winterhalter A, Bharadwaj M, Altman JD, Brooks A. Peptide-MHC class I tetrameric complexes display exquisite ligand specificity. *J Immunol.* 2000;165(11):6229-6234.
6. van Bergen CA, van Luxemburg-Heijs SA, de Wreede LC, Eefting M, von dem Borne PA, van Balen P, et al. Selective graft-versus-leukemia depends on magnitude and diversity of the alloreactive T cell response. *J Clin Invest.* 2017;127(2):517-529.
7. Koning MT, Kiełbasa SM, Boersma V, Buermans HPJ, van der Zeeuw SAJ, van Bergen CAM, et al. ARTISAN PCR: rapid identification of full-length immunoglobulin rearrangements without primer binding bias. *Br J Haematol.* 2017;178(6):983-986.
8. Lefranc MP, Giudicelli V, Ginestoux C, Bodmer J, Müller W, Bontrop R, et al. IMGT, the international ImMunoGeneTics database. *Nucleic Acids Res.* 1999;27(1):209-212.
9. Linnemann C, Heemskerk B, Kvistborg P, Kluin RJ, Bolotin DA, Chen X, et al. High-throughput identification of antigen-specific TCRs by TCR gene capture. *Nature medicine.* 2013;19(11):1534-1541.
